# Use of menopausal hormone therapy and risk of dementia: nested case-control studies using QResearch and CPRD databases

**DOI:** 10.1136/bmj.n2182

**Published:** 2021-09-30

**Authors:** Yana Vinogradova, Tom Dening, Julia Hippisley-Cox, Lauren Taylor, Michael Moore, Carol Coupland

**Affiliations:** 1 Division of Primary Care, School of Medicine, University of Nottingham, Nottingham, NG2 7RD, UK; 2 Mental Health and Clinical Neurosciences, School of Medicine, University of Nottingham, Nottingham, UK; 3 Nuffield Department of Primary Care Health Sciences, University of Oxford, Oxford, OX2 6GG, UK; 4 Primary Care Research Centre, Primary Care, Population Sciences and Medical Education (PPM), Faculty of Medicine, University of Southampton, Southampton, UK

## Abstract

**Objective:**

To assess the risks of developing dementia associated with different types and durations of menopausal hormone therapy.

**Design:**

Two nested case-control studies.

**Setting:**

UK general practices contributing to QResearch or the Clinical Practice Research Datalink (CPRD), using all links to hospital, mortality, and social deprivation data.

**Participants:**

118 501 women aged 55 and older with a primary diagnosis of dementia between 1998 and 2020, matched by age, general practice, and index date to 497 416 female controls.

**Main outcome measures:**

Dementia diagnoses from general practice, mortality, and hospital records; odds ratios for menopausal hormone treatments adjusted for demographics, smoking status, alcohol consumption, comorbidities, family history, and other prescribed drugs.

**Results:**

Overall, 16 291 (14%) women with a diagnosis of dementia and 68 726 (14%) controls had used menopausal hormone therapy more than three years before the index date. Overall, no increased risks of developing dementia associated with menopausal hormone therapy were observed. A decreased global risk of dementia was found among cases and controls younger than 80 years who had been taking oestrogen-only therapy for 10 years or more (adjusted odds ratio 0.85, 95% confidence interval 0.76 to 0.94). Increased risks of developing specifically Alzheimer’s disease were found among women who had used oestrogen-progestogen therapy for between five and nine years (1.11, 1.04 to 1.20) and for 10 years or more (1.19, 1.06 to 1.33). This was equivalent to, respectively, five and seven extra cases per 10 000 woman years. Detailed risk associations for the specific progestogens studied are also provided.

**Conclusion:**

This study gives estimates for risks of developing dementia and Alzheimer’s disease in women exposed to different types of menopausal hormone therapy for different durations and has shown no increased risks of developing dementia overall. It has shown a slightly increased risk of developing Alzheimer’s disease among long term users of oestrogen-progestogen therapies.

## Introduction

Menopause often manifests itself in a variety of both mental and physical symptoms, such as hot flushes, sleep disturbance, depression, or cognitive dysfunction, and about 80% of menopausal women are affected by such symptoms. Of these women, about 70% have symptoms that may also be associated with warnings of future neurological decline.^1^ Sex hormones, and particularly oestrogen, have been shown to have a neuroprotective effect, so declining concentrations of these may contribute to the development of neurodegenerative diseases.^2^ Although the severity of menopausal symptoms differs widely across the female population, the prescribing of oestrogen for at least some patients has been described as “appropriate and required.”^1^

Although menopausal hormone therapy (commonly known in the UK as hormone replacement therapy and hereafter referred to simply as hormone therapy) clearly eases menopausal symptoms, epidemiological evidence has been inconsistent regarding the effects of such treatments with respect to risks of developing dementia. Small studies using various cognitive and radiological measures have shown beneficial effects from hormone therapy.^3^ ^4^ However, the largest randomised controlled trial of hormone therapy, the Women’s Health Initiative Memory Study (WHIMS), which allocated postmenopausal women either to placebo or to conjugated equine oestrogen with or without medroxyprogesterone, showed an increased risk of dementia in both treated arms (although results were not statistically significant for oestrogen-only users).^5^ ^6^ A smaller trial, ELITE-Cog, reported no evidence of harm or of benefit on brain function from hormone therapy use.^7^ Two Finnish observational studies, based on national registries, have also provided conflicting results for risk of Alzheimer’s disease in hormone therapy users.^8^ ^9^ The first showed decreased risks for long term oestrogen use and no associations for long term oestrogen-progestogen use,^8^ whereas the second suggested increased risks for oestrogen treatments, with and without progestogen.^9^ The more recent study had some methodological flaws and a rather truncated study period, including only cases diagnosed up to 2013. Its results suggest possible increased risks of Alzheimer’s disease associated with any menopausal hormone therapy in addition to the known risks of venous thromboembolism or breast cancer. Given concerns expressed by some women’s health experts about potential problems caused by overemphasis on adverse risks from hormone therapy treatments,^10^ and the current National Institute for Health and Care Excellence (NICE) guideline stressing the need for more detailed information on side effects and adverse outcomes of hormone therapy,^11^ a confirmatory study investigating the findings is appropriate. Our previous studies on risks of venous thromboembolism and breast cancer have delivered useful and robust estimates of risk associations with different hormone therapy preparations, highlighting dydrogesterone as a potentially low risk progestogen.^12^ ^13^ In this study, we have aimed to provide similar detailed, accurate, and robust information with respect to use of hormone therapy and risk of dementia.

This study used a large data sample from primary care records to investigate the risks of developing dementia following hormone therapy use. It was designed with sufficient power not only to assess overall risk for women exposed to different types of long term hormone therapy but also to explore the differences between component hormones. The richness of the data available from two of the largest UK primary care databases (Clinical Practice Research Datalink (CPRD) and QResearch), together with linked hospital data, has allowed us to adjust for many more possible confounding factors, so offering new, more reliable estimates for doctors and their patients.

## Methods

### Study design

The published protocol contains full details of this study.^14^ In summary, we used two large UK primary care databases, QResearch and CPRD GOLD, to conduct two nested case-control studies. The data sources used were all general practices that had contributed to a database for more than 10 years. Each database provided an open cohort of all women aged over 55 and registered between 1 January 1998 and 31 July 2020. Women were excluded if, before their study entry, they had records of either dementia or dementia related prescriptions.

### Selection of cases and controls

For both databases, we identified all cases between 1 January 1998 and 31 July 2020 by using codes for dementia from patients’ clinical records or records of prescriptions for drugs used to treat dementia—donepezil, rivastigmine, memantine, and galantamine. For QResearch, general practice records, hospital episode statistics, and mortality data were all used. For CPRD, only a proportion of practices (45%) were linked to hospital and mortality data, so we defined cases from unlinked practices by using only general practice records.

Dementia is usually diagnosed in secondary care memory clinics staffed by specialists or in general practices following a set of guideline investigations including computed tomography scans and supported by specialists. Where patients present late with evident moderate or advanced dementia, and where circumstances mean that a patient is unlikely to benefit from referral to or review by specialists in a memory clinic, the diagnosis may be made by the general practitioner on the basis of clinical findings. Whatever the process, diagnostic information is available within the practice responsible for entering the information.

The type of dementia, however, was not always available or may not have been transferred. The data showed that this was more pronounced earlier in the study period and among older patients, probably because of improvements in the diagnostic process over time and differing circumstances of patients at the time of diagnosis (supplementary figure A).

Using incidence density sampling,^15^ we matched cases by year of birth to up to five controls—women from the same practice but without a diagnosis of dementia at the time of diagnosis of their case (index date). We included only those cases and controls with at least 10 years of medical records before the index date. No overlap existed between the two sets of cases and controls, because a patient can be registered with only one practice and QResearch and CPRD GOLD receive data from practices using different computer systems.

### Exposure to hormone therapy

We extracted all prescriptions indicated for menopausal treatment for systemic oestrogen and progestogen (oral, subcutaneous, or transdermal). We assessed the prevalence rate of prescribing of hormone therapy by general practitioners from the CPRD study population. For each study year, we divided the number of women with at least one prescription for hormone therapy by the total number of women, all being registered for the whole year of interest.

Early symptoms of dementia before diagnosis, such as sleep problems or depression, may be taken for menopause symptoms, and cognitive decline may also be associated with a cessation of menopausal hormone therapy. So, to minimise possible protopathic bias, we excluded prescriptions for hormone therapy issued in the last three years before the index date from our main analysis.^16^ However, we also ran a sensitivity analysis using all records up to one year before the index date, to check whether any under-reporting bias had been introduced by this exclusion of prescriptions.

We took a first prescription for systemic oestrogen as the start of exposure to hormone therapy. Patients with no prescriptions containing a progestogen after this date were classified as users of an oestrogen-only therapy. Patients with any subsequent prescription containing a progestogen were classified as combined therapy users. We included prescriptions for tibolone and topical hormonal preparations (vaginal pessaries or cream), because these are commonly prescribed to menopausal women.

To account for switching between hormonal therapies, we analysed exposures to different preparations separately. For oestrogen-only users, we considered two types of oestrogen (conjugated equine oestrogen and estradiol), two routes of application (oral or transdermal/injection) and two dosage levels—low (defined as ≤0.625 mg/day for oral conjugated equine oestrogen, ≤1 mg/day for oral estradiol, and ≤50 mg for transdermal oestradiol) and high (all other doses). For oestrogen-progestogen users, we focused on progestogen types (norethisterone acetate, levonorgestrel, medroxyprogesterone, and dydrogesterone) and did not distinguish between oestrogen types. Norethisterone and dydrogesterone, however, were prescribed only with estradiol and medroxyprogesterone, and levonorgestrel was prescribed mostly with conjugated equine oestrogen.^13^ Some oestrogen-progestogen users had records including intervals of oestrogen-only therapy use, so we adjusted for these oestrogen-only exposures.

We calculated durations of exposure by summing prescription periods (including gaps between prescriptions of <90 days) and categorised them as never, <1 year, 1 to <3 years, 3 to <5 years, 5 to <10 years, and ≥10 years. We categorised the time interval between the index date and the last prescription (more than three years before the index date) as between 3 and <5 years, 5 to <10 years, and ≥10 years. To assess whether associations between hormone therapy and risk of dementia depend on age at starting hormone therapy, we also separately analysed exposures to hormone therapy started before the age of 60 and those started at or after 60. For all treatments, we used no exposure more than three years before the index date as the reference category.

### Confounders

We adjusted the analyses for indications for hormone therapy use and factors associated with risk of dementia, which might have influenced a doctor’s prescribing decisions.^17^ We extracted these if they were recorded at least 10 years before the index date to make them closer to the likely time of hormone therapy use. We also did a sensitivity analysis using confounders recorded up to three years before the index date. The confounders are listed in table 1 and include lifestyle factors; self-assigned ethnicity; family history of dementia; records of early menopause; oophorectomy/hysterectomy; menopausal symptoms; comorbidities, including chronic conditions; and use of other relevant drugs (if prescribed at any time more than 10 years before the index date).

**Table 1:** Characteristics of cases with dementia and matched controls, 10 years before index date, by database (QResearch and CPRD). Values are percentages (numbers) unless stated otherwise

Characteristics	QResearch			CPRD	
	Cases (n=68 738)	Controls (n=267 490)		Cases (n=49 763)	Controls (n=229 926)
Mean (SD) age, years	83.8 (6.6)	83.5 (6.3)		83.0 (7.5)	82.6 (7.3)
Age group, years:					
55-64	0.2 (138)	0.2 (552)		1.9 (958)	2.0 (4500)
65-74	8.7 (5995)	8.8 (23 518)		10.9 (5417)	11.3 (25 890)
75-84	42.6 (29 255)	44.6 (119 413)		41.4 (20 616)	43.2 (99 347)
85-110	48.5 (33 350)	46.4 (124 007)		45.8 (22 772)	43.6 (100 189)
Mean (SD) years of records	16.5 (4.4)	16.5 (4.4)		14.9 (4.1)	15.4 (4.3)
Ethnicity:					
White	70.6 (48 521)	72.7 (194 586)		64.9 (32 296)	63.4 (145 691)
Not recorded	26.0 (17 893)	24.3 (64 983)		34.0 (16 941)	35.7 (82 078)
Bangladeshi	0.2 (110)	0.1 (335)		<0.1 (15)	<0.1 (11)
Black African	0.2 (150)	0.2 (523)		<0.1 (16)	<0.1 (88)
Caribbean	1.2 (852)	0.9 (2387)		0.2 (113)	0.2 (392)
Chinese	0.1 (51)	0.1 (305)		<0.1 (21)	0.1 (141)
Indian	0.7 (470)	0.7 (1988)		0.2 (110)	0.2 (492)
Other	0.5 (347)	0.4 (1141)		0.4 (176)	0.3 (677)
Other Asian	0.2 (154)	0.2 (605)		0.1 (29)	0.1 (211)
Pakistani	0.3 (190)	0.2 (637)		0.1 (46)	0.1 (145)
Fifth of Townsend deprivation*:				(n=26 387)	(n=120 315)
1=most affluent	28.1 (19 293)	30.1 (80 449)		22.3 (5878)	23.7 (28 557)
2	25.2 (17 322)	25.7 (68 878)		23.9 (6301)	24.4 (29 378)
3	21.5 (14 781)	20.8 (55 771)		21.7 (5723)	21.7 (26 051)
4	15.7 (10 772)	14.7 (39 251)		19.9 (5259)	18.9 (22 773)
5=most deprived	9.6 (6570)	8.7 (23 141)		12.2 (3226)	11.3 (13 556)
Body mass index:					
Recorded	73.2 (50 302)	72.5 (194 020)		77.0 (38 311)	76.6 (176 054)
Mean (SD)	26.7 (4.9)	26.9 (4.8)		27.2 (4.9)	27.3 (4.8)
Smoking:					
Recorded	79.8 (54 853)	79.0 (211 450)		85.9 (42 726)	85.0 (195 355)
Non-smoker	52.7 (36 235)	53.7 (143 671)		59.7 (29 724)	61.2 (140 763)
Ex-smoker	17.7 (12 163)	17.0 (45 365)		17.0 (8458)	15.7 (36 120)
Light (1-9 cigarettes/day)	7.2 (4947)	6.4 (17 205)		3.9 (1953)	3.5 (8060)
Moderate (10-19/day)	1.5 (1046)	1.4 (3640)		3.3 (1660)	3.0 (6907)
Heavy (≥20/day)	0.7 (462)	0.6 (1569)		1.9 (931)	1.5 (3505)
Alcohol consumption:					
Recorded	73.2 (50 327)	72.5 (194 027)		77.0 (38 313)	75.9 (174 560)
None	50.5 (34 721)	49.2 (131 524)		37.5 (18 638)	35.5 (81 517)
Trivial (<1 units/day)	15.8 (10 887)	16.3 (43 483)		23.5 (11 683)	23.8 (54 700)
Light (1-2 units/day)	4.5 (3065)	4.7 (12 610)		9.8 (4856)	10.3 (23 744)
Moderate (3-6 units/day)	2.3 (1599)	2.3 (6225)		5.8 (2865)	5.9 (13 461)
Heavy (7-9 units/day)	0.1 (41)	0.1 (149)		0.4 (208)	0.4 (894)
Very heavy (≥10 units/day)	<0.1 (14)	<0.1 (36)		0.1 (63)	0.1 (244)
Chronic conditions:					
Anxiety	8.1 (5550)	7.0 (18 639)		13.0 (6481)	11.3 (25 989)
Cancer	6.2 (4281)	6.2 (16 584)		5.8 (2909)	6.0 (13 784)
Coronary heart disease	11.1 (7663)	9.6 (25 780)		12.6 (6251)	10.8 (24 722)
Depression	17.9 (12 319)	14.8 (39 570)		21.6 (10 725)	18.0 (41 500)
Diabetes	8.7 (5948)	6.5 (17 378)		8.2 (4074)	6.0 (13 753)
Hearing loss	5.4 (3696)	4.9 (13 091)		9.9 (4935)	8.8 (20 170)
Hypertension	41.4 (28 484)	40.2 (107 524)		41.1 (20 454)	40.0 (91 877)
Parkinson's disease	0.5 (351)	0.2 (571)		0.6 (313)	0.2 (560)
Stroke	5.2 (3557)	3.9 (10 441)		5.6 (2779)	4.2 (9733)
Other characteristics:					
Early menopause	9.0 (6182)	8.7 (23 152)		7.8 (3871)	7.4 (17 023)
Hysterectomy/oophorectomy	21.9 (15 020)	21.7 (58 079)		20.2 (10 071)	19.8 (45 446)
Menopausal symptoms	13.3 (9158)	13.2 (35 197)		15.3 (7637)	15.8 (36 309)
Family history of dementia	0.2 (121)	0.1 (192)		0.1 (54)	<0.1 (111)
Any use of other drugs before index date:					
Anticholinergics	45.8 (31 499)	41.6 (111 367)		48.8 (24 282)	44.8 (103 089)
Antiarrhythmics	0.1 (88)	0.1 (337)		0.1 (67)	0.1 (334)
Antidepressants	23.3 (15 998)	19.8 (52 858)		23.5 (11 685)	20.0 (46 059)
Antiepileptics	2.2 (1538)	1.9 (5058)		2.7 (1319)	2.2 (5135)
Antihistamines	9.6 (6618)	8.7 (23 277)		11.1 (5530)	10.4 (23 945)
Antimuscarinics	5.1 (3496)	4.0 (10 653)		5.6 (2791)	4.3 (9962)
Antiparkinsonian drugs	0.6 (409)	0.3 (881)		0.7 (360)	0.3 (750)
Antipsychotics	2.8 (1946)	2.0 (5339)		3.0 (1481)	2.1 (4933)
Antispasmodics	6.8 (4650)	6.3 (16 754)		7.9 (3923)	7.7 (17 734)
Antivertigo drugs	18.8 (12 897)	17.3 (46 224)		21.1 (10 514)	19.9 (45 673)
Bronchodilators	3.6 (2443)	3.1 (8192)		3.8 (1890)	3.2 (7375)
Muscle relaxants	0.6 (431)	0.6 (1517)		1.1 (566)	1.1 (2537)
Antihypertensives	55.7 (38 315)	53.0 (141 821)		57.2 (28 452)	54.4 (125 180)
Clonidine	2.0 (1343)	1.9 (5133)		3.1 (1522)	3.1 (7096)
Benzodiazepines	12.6 (8694)	10.9 (29 288)		15.5 (7716)	13.8 (31 821)
Statins	22.2 (15 292)	19.2 (51 379)		22.2 (11 061)	19.4 (44 561)

CRPD=Clinical Practice Research Datalink.

* Based on linked practices.

### Statistical analysis

We ran data extraction, processing, and analysis separately for QResearch and CPRD. The analyses were as similar as the datasets permitted. We assessed risks of dementia associated with hormone therapy exposures by using conditional logistic regression and presented them as odds ratios with 95% confidence intervals. A small proportion of data for body mass index, smoking, and alcohol consumption were missing (table 1). We compared the patterns of missingness and assumed missingness at random, and we then imputed the values by using chained equations over 10 imputed datasets. The imputation model included all listed confounders, exposures, and case-control indicators, and we combined the odds ratios obtained from the imputed datasets by using Rubin’s rule.^18^

We used a meta-analysis technique to combine the odds ratios obtained from the analyses of QResearch and CPRD.^19^ Because the data were collected in the same setting and processed and analysed as similarly as possible, we used a fixed effect model with inverse variance weights to combine the results, checking for any heterogeneity with a sensitivity analysis using a random effect model. Only combined results appear in the main figures and text; the separate QResearch and CPRD results can be found in the supplementary materials.

To estimate absolute magnitudes of dementia risk for women in different exposure categories, we calculated rates and numbers of extra cases, determining the incidence rate in the unexposed female population from CPRD data and using combined odds ratios from the reported analyses.^20^

We used Stata 16 for all analyses. We chose a 1% level of statistical significance to allow for multiple comparisons, but we have presented 95% confidence intervals to facilitate comparison with other studies.

### Additional analyses

Hormone therapy was not widely prescribed until the late 1980s,^21^ so older women in the cohort might have had less exposure. To account for this possible inconsistency, we analysed women younger than 80 years at their date of diagnosis of dementia or index date and their controls separately from women aged 80 or older and their controls.

We also analysed separately the two most common subgroups of cases and their controls—the first restricted to cases of Alzheimer’s disease and the second to cases of vascular dementia. This provided estimates of the specific risks associated with hormone therapy use of developing these dementia types and facilitated comparison with other studies. Owing to the low numbers of exposed cases, only main exposure types could be considered.

The results from the subgroup analysis restricted to cases younger than 80 years old and to cases with Alzheimer’s disease suggested a possible relation between continuous durations of exposure to hormone therapy and risk of developing the disease. So we used fractional polynomials to model the associations,^22^ selected the linear relation suggested, and ran these analyses using continuous exposure—separately for each database and combining the coefficients by using the meta-analysis technique described earlier.

We used all available records for the main analysis. As only about 45% of CPRD practices had links to Hospital Episode Statistics, Office for National Statistics mortality data, and patient level Townsend deprivation index, we repeated the CPRD analysis on the subgroup of patients with fully linked data. We also ran sensitivity analyses for both databases omitting cases identified only by prescription for a dementia drug. Some women might have been exposed to hormone therapy before registering with their practice or before the practice installed software to digitise paper records, so we repeated these analyses using subgroups of women with data available from before or at their 50th birthday to investigate the possible effects of some women having unrecorded earlier exposures. Finally, we repeated the analysis using only cases and controls with no missing data for body mass index, smoking, or alcohol consumption.

### Patient and public involvement

This study was initiated because NICE has noted a need for more detailed information related to hormone therapy use and dementia, recommending further research.^11^ Because dementia is a relatively rare condition, we used routinely collected data and well established statistical techniques. Patients were, therefore, not involved in setting the research question or outcome measures and did not help to develop the study design. While working on previous projects on the safety of hormone therapy with respect to risks of breast cancer and venous thromboembolism,^12^ ^13^ however, we had formal and informal conversations with menopausal and postmenopausal women. In general, the women stressed the importance for them of access to hormone therapy, and all users showed high adherence to their medications. The first author is also a regular attendee at a Menopause Café event at Nottingham University, which facilitates both the acquisition of useful information about women’s experience of menopause and dissemination of our research findings.

## Results

### Sample description

Overall, we identified 118 501 cases across the two databases (68 738 from QResearch and 49 763 from CPRD) and matched them to 497 416 controls (supplementary figure B). Of these, 4819 (4%) cases did not have any clinical records of dementia but had prescriptions for antidementia drugs. The proportion of cases with dementia data only in their hospital or mortality records differed between the databases—7091 (10%) for QResearch cases and 7942 (30%) for the 26 421 linked CPRD cases.

Table 1 shows the descriptive statistics for cases and controls separately for QResearch and CPRD, showing the general similarity between the databases. Across the databases, the mean age of cases was 83.5 (SD 7.0) years, and the mean number of years of recorded clinical and prescription data before the index date was 16.0 (4.3) years for cases and 15.8 (4.2) years for controls. Cases had records for mental health conditions (anxiety, depression) slightly but consistently more often than did controls and had more records for other general health conditions (coronary heart disease, diabetes, hearing loss, hypertension, and Parkinson’s disease). Cases were also more likely than controls to be given prescriptions for anticholinergics, antihypertensives, benzodiazepines, and statins (table 1).

### Exposure to hormone therapy

**Fig 1:**
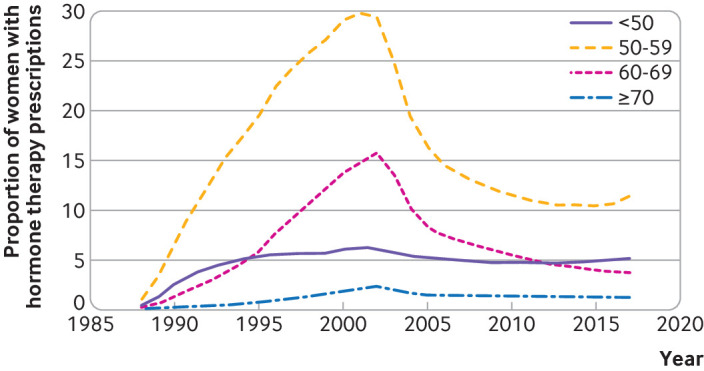
Proportion of women exposed to hormone therapy by age over study period in Clinical Practice Research Datalink

Figure 1 shows the prevalence rate for prescribing of hormone therapy from the earliest available records in CPRD to three years before the end of the study period. The data show a prevalence rate for hormone therapy use of 1% in 1988, rising consistently to reach a peak by 2000 (30% for women between 50 and 59 years of age) and then falling back after 2003, the lowest subsequent rate being 10% in 2013 for women aged between 50 and 59 years.

Overall, up to three years before the index date, 16 291 (13.7%) cases and 68 726 (13.8%) controls had been exposed to hormone therapy. Women with prescriptions for hormone therapy were, in general, younger and more likely to live in an affluent area. They more often had a diagnosis of anxiety or depression—38% of hormone therapy users versus 23% of never users in cases, and 32% of users versus 19% of never users in controls. Oestrogen-progestogen users had better general health than never users or oestrogen-only users and had lower prevalence of coronary heart disease, stroke, diabetes, and hypertension. Users of hormone therapy were also more likely to be treated with anticholinergics (consistent across most types) and benzodiazepines (supplementary table A).

### Main analysis

**Fig 2:**
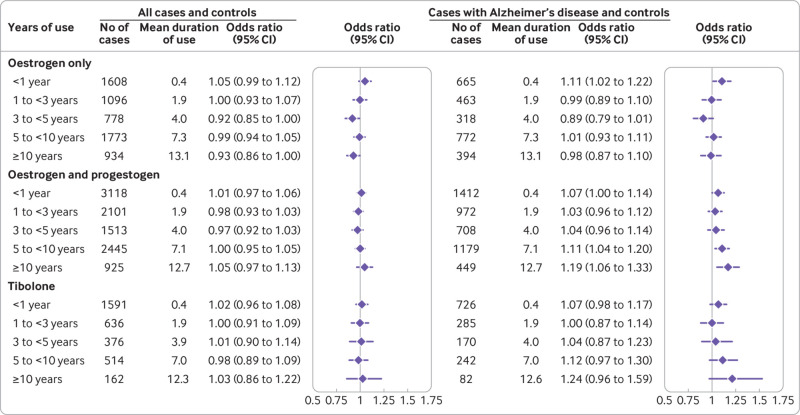
Use of oestrogen only, oestrogen-progestogen, and tibolone and adjusted odds ratios for dementia overall and for Alzheimer’s disease. Odds ratios are adjusted for smoking, alcohol consumption, Townsend fifth (QResearch only), body mass index, ethnicity, family history of dementia, oophorectomy/hysterectomy, records of menopause, comorbidities, other drugs, and years of data. Cases were matched to controls by age, general practice, and index date.

**Fig 3:**
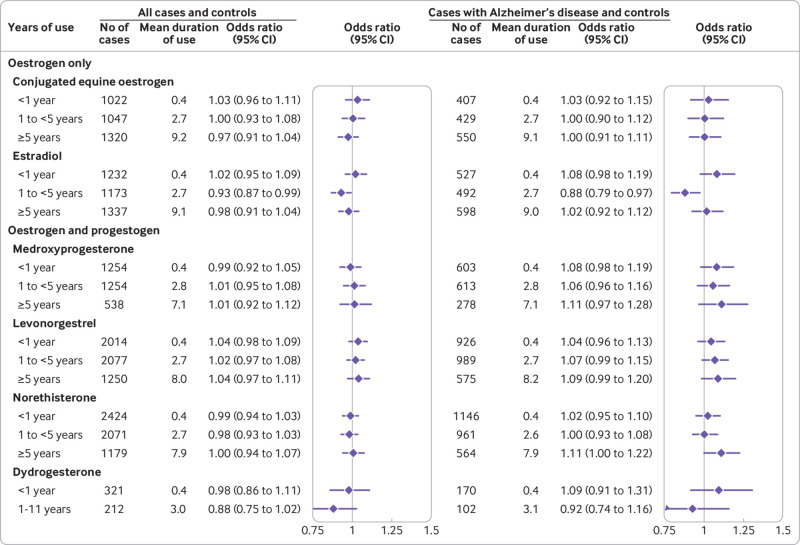
Use of different hormones and adjusted odds ratios for dementia overall and for Alzheimer’s disease. Odds ratios are adjusted for smoking, alcohol consumption, Townsend fifth (QResearch only), body mass index, ethnicity, family history of dementia, oophorectomy/hysterectomy, records of menopause, comorbidities, other drugs, and years of data. Cases were matched to controls by age, general practice, and index date

The “unadjusted” analysis (accounting only for matching of age and general practice) showed small increased risks of developing dementia associated with hormone therapy use (supplementary table B). After adjusting for the full range of available confounders, however, we found no statistically significant overall associations between use of hormone therapy and risk of dementia either for oestrogen-only treatments (adjusted odds ratio 0.99, 95% confidence interval 0.96 to 1.02) or for oestrogen-progestogen treatments (1.00, 0.97 to 1.03). This finding was independent of the length of exposure to hormone therapy and of the length of time after discontinuation of treatment. The finding was also consistent for all different hormone types used in the preparations, with slightly lower risks for oestrogen-dydrogesterone taken for between one and 11 years (adjusted odds ratio 0.88, 0.75 to 1.02) (fig 2; fig 3; supplementary tables C-F). Analysis of risks associated with the age at which hormone therapy had been started (at or after 60 years, or before 60) also showed no statistically significant associations (supplementary figure C).

### Additional analyses

#### By age at diagnosis (<80 and ≥80 years)

Among women with a diagnosis of dementia, 27% had received their diagnosis before the age of 80. The patterns of confounders relating to lifestyle (social deprivation, smoking, and alcohol consumption) were similar in both the younger and older subgroups. Younger women had fewer records for most comorbidities related to general health but more records related to diabetes and mental health problems (anxiety or depression).

**Fig 4:**
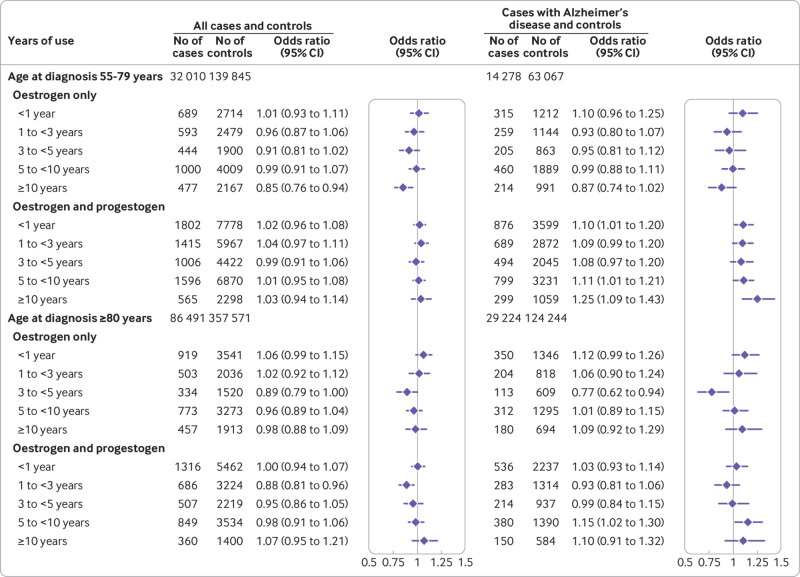
Use of oestrogen only, oestrogen-progestogen, and tibolone in women of different ages and adjusted odds ratios for dementia overall and for Alzheimer’s disease. Odds ratios are adjusted for smoking, alcohol consumption, Townsend fifth (QResearch only), body mass index, ethnicity, family history of dementia, oophorectomy/hysterectomy, records of menopause, comorbidities, other drugs, and years of data. Cases were matched to controls by age, general practice, and index date

In the younger group, 30% of cases and 29% of controls had prescriptions for hormone therapy, but only 8% of the older group had ever used hormone therapy. In general, the analyses for risk of dementia in both subgroups gave results similar to the main analysis. In the younger group, however, exposure to oestrogen-only treatment for more than 10 years was associated with some decreased risk (adjusted odds ratio 0.85, 0.76 to 0.94; P=0.003), and a linear relation between duration of the exposure and risk of dementia suggested a 1.1% decrease per year of hormone therapy use (fig 4; supplementary table G).

#### By type of dementia (Alzheimer’s disease or vascular dementia)

Overall, 39 876 (34%) of patients with dementia had a diagnosis of Alzheimer’s disease alone and 24 867 (21%) had a diagnosis of vascular dementia alone, with 3626 (3%) having diagnoses of both (cases with both diagnoses were added to both subgroups before analysis). Cases with a diagnosis of Alzheimer’s disease were slightly younger than cases with vascular dementia, having respectively a mean age of 82.2 (SD 6.9) and 83.4 (6.6) years. Cases with Alzheimer’s disease had lower use of other drugs and slightly better physical and mental health than cases with vascular dementia.

For oestrogen-only users, an increased risk of developing Alzheimer's disease was seen only in the shortest exposure category of less than one year (adjusted odds ratio 1.11, 1.02 to 1.22), with no associated risks for longer term exposures. For oestrogen-progestogen users, however, we found statistically significantly increased risks for the longer exposures (adjusted odds ratio 1.11 (1.04 to 1.20) for five to nine years; 1.19 (1.06 to 1.33) for ≥10 years) (supplementary table H). We found a linear relation between duration of exposure to oestrogen combined with any progestogen and risk of Alzheimer’s disease, suggesting that risk of developing the disease may increase by 1.2% per year of hormone therapy use (supplementary figure D).

Analysis of the different hormones showed similar relations with risk of Alzheimer’s disease for conjugated equine oestrogen and estradiol and between most types of progestogen, but no findings reached statistical significance. Although oestrogen-dydrogesterone seemed to be associated with the lowest risks among oestrogen-progestogen combinations, the confidence intervals were too broad for definite conclusions to be drawn. (fig 3; supplementary table H).

Within the subgroup of women with a diagnosis of Alzheimer’s disease, the differences in risk between the component subgroup of women given a diagnosis when younger than 80 years and the component subgroup of those given a diagnosis at 80 or older are shown in figure 4. The patterns are similar to those for the complete subgroup, but the confidence intervals are wider for the older group. Risks after discontinuation remained increased for those oestrogen-progestogen users with a final prescription between three and 10 years before the index date (supplementary table H). Analysis of component subgroups based on hormone therapy initiation at different ages (before 60 years, or at or after 60 years) showed no differences in associated risks (supplementary figure C). Analyses of cases with vascular dementia and their controls showed no increase in risks (supplementary table H).

#### Sensitivity analyses

The subgroups of cases and controls registered before their 50th birthday represented about 2% of the main sample (942 cases from QResearch and 1689 cases from CPRD). By contrast with the full samples used in the main and other analyses, these subsamples showed some differences in make-up between the databases—on average, cases from QResearch were older than those from CPRD (respectively 71.1 (SD 4.4) and 65.4 (6.0) years), and the proportion of women exposed to hormone therapy was higher in QResearch (53% in cases and controls versus 49% in cases and 46% in controls in CPRD). Associated risks also seemed to differ between the databases. In the QResearch subgroup, we found no statistically significant associations between exposure to hormone therapy and risk of dementia. In the CPRD subgroup, however, although the association for oestrogen-only therapy (adjusted odds ratio 1.18, 0.91 to 1.53) did not reach a statistically significant level because of the small numbers involved, the risk for oestrogen-progestogen users of developing dementia seemed to be increased regardless of duration of exposure (1.40, 1.22 to 1.61) (supplementary table I). All the results from other sensitivity analyses designed to investigate the effects of different limitations (described in the protocol,^14^ and referred to in Methods above) were similar to the main findings.

#### Extra cases in exposed women

Per 10 000 woman years, the overall rate of Alzheimer’s disease for women older than 55 who had no records of prescriptions for hormone therapy was 39.4. By the same measure, the estimated rate of Alzheimer’s disease in women who took combined oestrogen-progestogen hormone therapy for between five and nine years was 43.9 (95% confidence interval 40.9 to 47.2), an extra 4.5 (1.5 to 7.7) cases, and the estimated rate for women who used combined therapy for ≥10 years was 46.9 (42.0 to 52.3), an extra 7.4 (2.5 to 12.9) cases. For women aged between 55 and 79, the rate of Alzheimer’s disease was 14.3 per 10 000 woman years, and the estimated rate for women with prescriptions for combined therapy for ≥10 years was 17.9 (15.6 to 20.5), an extra 3.6 (1.3 to 6.2) cases.

## Discussion

This large observational study found no overall association between use of menopausal hormone therapy and risk of developing dementia. This finding was consistent across different types of hormones, doses, applications, and time of hormone therapy initiation. We found a decreased risk dementia for cases and controls younger than 80 years at diagnosis who had been taking oestrogen-only therapy for 10 years or more. However, a subgroup analysis of cases with a diagnosis of Alzheimer’s disease showed a small increase in risk associated with oestrogen-progestogen therapy. This rose with each year of exposure, reaching average risk increase of 11% for between five and nine years of use and 19% for 10 years or more—equivalent to, respectively, five and seven extra cases per 10 000 woman years.

### Strengths and weaknesses of study

The main strengths of this study were a very large sample representative of the general population and a study design that captured all known cases and used the richness of the data and precision of recording for prescribed drugs. The size of the sample allowed us to investigate the risks for specific treatment types and the effects of durations of exposure. We were also able to explore risks for specific subgroups of women and the effects of age at start of the therapy. The similarity of the results obtained from two databases containing data collected using different software and the results from our various sensitivity analyses have also shown the robustness of our findings.

The main weakness of our study was a possible lack of available data before the index date for some older women, whose menopause started before their registration or before collection of these data by their practice. We consider, however, that these women were unlikely to be greatly exposed to hormone therapy because the rate of prescribing was very low 10 years before the start of the study and our prescribing rate estimation taken from the CPRD database is similar to a rate based on government prescription data covering the period between 1988 and 1994.^21^ We also explored the possible effects of such under-calculations of exposure by analysing separately cases based on women with a diagnosis before their 80th birthday and those with a diagnosis after that. Younger women were more likely to have fully recorded data preceding their menopause, and the results obtained from both of these subgroup analyses were consistent with those from our main analysis, which suggests that the possible lack of data for some older women has had little effect.

Our main analysis includes all cases of dementia, irrespective of type of dementia, as more than half the sample had no dementia type specified in their general practice records. When we considered the subsamples of patients with a recorded diagnosis of Alzheimer’s disease or of vascular dementia, about 10% of cases of Alzheimer’s disease (15% for vascular dementia cases) also had a recorded diagnosis for the other type, showing that mixed forms of dementia are not uncommon. Our analyses found no association between hormone therapy and dementia risk for the group with dementia as a whole or for patients who developed vascular dementia. However, we found some increased risk within the subgroup based on patients with a diagnosis of Alzheimer’s disease. One of the strengths of this study is that the sample size was large enough to show these different patterns of risk. However, many of the patients with a non-specific diagnosis of dementia will actually have had Alzheimer’s disease or mixed Alzheimer’s-vascular dementia, so we can presume that the risks of developing Alzheimer’s disease are very unlikely to be higher than our estimates, and they could be somewhat lower.

Although levels of completeness within the databases are quite high for diagnoses, onset and symptoms of menopause are not consistently recorded. Even in the groups of women with oestrogen-progestogen prescriptions, only 48% of women had records of menopausal symptoms compared with an expected 80%.^1^ Because some symptoms of menopause are similar to symptoms of developing dementia, women without menopausal symptoms may have different underlying risk associations for development of dementia. In our sample, most of them fall within the category of non-users (in our unexposed groups the level of recording of menopausal symptoms was only 10%). Comparing women with and without symptoms may, therefore, shift odds ratios away from unity, but our adjustment for records of menopausal symptoms will have reduced that shift.

Because psychological and mental health related symptoms of menopause can be early signs of developing dementia, using prescriptions and confounders from close to the time of diagnosis may introduce confounding by indication bias. Our design reduced this possibility by excluding prescriptions for hormone therapy issued within the three years before the index date and records of confounders within the previous 10 years.

As with any study based on routinely collected data, our study had a small proportion of women with missing data for body mass index, smoking, and alcohol consumption. We overcame this limitation by using multiple imputations for missing values. Not all known risk factors for dementia were available, so we could not adjust for level of education, for physical or mental inactivity, or for social isolation.^17^ How the lack of data for these confounders may have influenced our estimates of the associations between hormone therapy and dementia risk is not clear, so some residual confounding bias may be present.

### Strengths and weaknesses in relation to other studies

Associations between increased risk of developing types of dementia and oestrogen menopausal therapies have been widely studied, but, despite broad agreement in many areas, all studies to date show various weaknesses in terms of coverage, data completeness, or methodological consistency. Risk of Alzheimer’s disease associated with oestrogen menopausal therapy has, for example, has been reported as decreased in a recent meta-analysis of 21 studies (odds ratio 0.63, 0.58 to 0.80), but the included studies were small and heterogeneous in design, and none reported on use of progestogens.^23^ The largest trial, WHIMS, based on 40 dementia cases, showed an increased risk of developing dementia for oestrogen-progestogen treatments (hazard ratio 2.05, 1.21 to 3.48) but not for oestrogen-only treatments.^5^ ^6^ Although broadly in line with our results, the WHIMS follow-up periods were considerably shorter (only 5.2 years for the oestrogen-only arm and 4 years for the oestrogen-progestogen arm) than we have achieved with the data available to our study, and the only hormonal types included were conjugated equine oestrogen and medroxyprogesterone. The WHIMS trial also investigated the effect of hormone therapy from the age of 65, but some of the included women had used hormonal therapy before entering (45% for the oestrogen-only arm and 22% for the oestrogen-progestogen arm). Although this information was used in the analysis, clarity about how this was done is lacking.

Two large observational studies, both focusing only on cases with Alzheimer’s disease, have been based on data from the Finnish national registries.^8^ ^9^ The more recent of these highlighted increased risk associations both for oestrogen-only and oestrogen-progestogen therapies.^9^ The number of cases included in the study was twice as large as ours (84 739 *v* 43 502), but the period for which patient data were available was much shorter (1994-2013 *v* 1988-2020). The study also had some possibly important methodological weaknesses—all exposure up to the point of diagnosis was included, with no exclusion of any exposure records within a period immediately before diagnosis to avoid protopathic bias. Each case was matched to only one control, which is not adequate given the low prevalence of some exposures. Finally, menopausal symptoms and other important confounders relating to hormone therapy use, such as mental health problems and use of other drugs, were not used to adjust the study results. As confirmed by the estimates from our unadjusted analysis, this is likely to have produced higher odds ratios.

The earlier study, from a different Finnish team but based on the same data source, seems to have had a methodologically more sound study design. It included four controls per case, used linkages to hospital data for extracting information for confounding factors, and excluded data in the last five years before the index date.^8^ However, this study had access to data over an even shorter time period, ending in 2011, two years earlier than the later study.^9^ The results for exposure for 10 years or more showed no associations with Alzheimer’s disease (odds ratio 0.91 (0.84 to 0.99) for oestrogen-only and 1.05 (0.90 to 1.22) for oestrogen-progestogen). The study, however, showed increased risks for shorter term exposures to oestrogen with or without progestogen. Our view is that the estimates of both this and the other study may have been affected by the relative lack of longer term historical data (before 1994), making the numbers for long term exposure quite small. In the first study, this probably led to some under-ascertainment of exposure in the model; in the second study, we suspect that methodological weaknesses may have affected the detailed results in unpredictable ways.

### Meaning of study: explanations and implications

Biological studies have suggested possible neuroprotective effects of oestrogen on the brain.^1^ ^2^ For long term exposure (≥10 years) in women younger than 80 at the time of diagnosis (adjusted odds ratio 0.85, 0.76 to 0.94), and to a lesser extent in the subgroup analysis relating to Alzheimer’s disease (0.87, 0.74 to 1.02), the results from our main analysis also supported possible protective effects for oestrogen-only therapies. For women 80 years or older at the time of diagnosis, we found no such associations, probably because of the prevailing low rates of prescription at the time of their menopause and, perhaps, also because of a decrease in the number of oestrogen receptors with age. Also according to biological studies, progestogen administered with oestrogen may result in the opposite of a protective effect because it can counteract the effects of the oestrogen.^1^ ^2^ This would be consistent with our findings, which show an increase in risk of developing Alzheimer’s disease risk for long term oestrogen-progestogen usage, particularly among younger women (1.25, 1.09 to 1.43).

This study has shown that women taking oestrogen-only therapies are not at greater risk of developing Alzheimer’s disease and dementia overall, but that the risk of developing Alzheimer’s disease is increased among women with long term exposure of more than five years to oestrogen-progestogen therapies. These associations do not prove any causal link, but risks of breast cancer are also associated with longer term hormone therapy use, so the results are in line with existing concerns in guidelines about long term exposures to combined hormone therapy treatments.^24^

### Conclusions

This large study of the general female population has used recently collected clinical and prescribing data and reports no overall risks of developing dementia associated with use of menopausal hormone therapy, consistent across types of treatment and durations, age categories, and times of therapy initiation. The study, however, did find associations with increased risk of developing Alzheimer’s disease among long term users of oestrogen-progestogen therapies. The findings will be helpful to policy makers, doctors, and patients when making choices about hormone therapy.

## What is already known on this topic

Laboratory studies and small trials have suggested a beneficial link between use of menopausal hormone therapy and age related brain decline
The Women’s Health Initiative Memory Study, however, found an increased risk of developing dementia among users of oestrogen-progestogen treatments
A recent large (but methodologically flawed) observational study flagged an increased risk of developing Alzheimer’s disease among users of oestrogen-only and oestrogen-progestogen treatments

## What this study adds

For all commonly prescribed menopausal hormone treatments in the UK, no increased risk associations were seen for the development of dementia globally
Specifically for Alzheimer’s disease, a small increased risk was associated with more than five years of use of oestrogen-progestogen treatments
This large observational study also provides the most detailed estimates of risk for individual treatments

## Data Availability

To guarantee the confidentiality of personal and health information, only the authors have had access to the data during the study in accordance with the relevant licence agreements. Access to the QResearch data is according to the information on the QResearch website (www.qresearch.org). CPRD linked data were provided under a licence that does not permit sharing.

## Web extra

Extra material supplied by authors

Web appendix: Supplementary materials

## Notes

Contributors: YV led the work of the study team, contributed to development of the study protocol, reviewed the literature, designed the study, organised the extraction of CPRD data, did the analysis on both datasets, and wrote all drafts of the manuscript. JHC initiated the study, did the original literature review, drafted the original study protocol, and organised the extraction of the QResearch data. TD provided expertise on dementia and Alzheimer’s disease. MM, JHC, and LT advised on clinical aspects related to primary care and interpretation of the results. CC contributed to the development of the idea and the study design and to the analysis. All co-authors critically reviewed the paper and approved the submitted version. YV is the guarantor. The corresponding author attests that all listed authors meet authorship criteria and that no others meeting the criteria have been omitted.

The lead author affirms that the manuscript is an honest, accurate, and transparent account of the study being reported; that no important aspects of the study have been omitted and that any discrepancies from the study as planned (and, if relevant, registered) have been explained.

Dissemination to participants and related patient and public communities: We used anonymised data and, therefore, did not have direct contact information for individual study participants for dissemination of the results. The results will be presented in a lay summary and sent to NIHR SPCR and QResearch.

Provenance and peer review: Not commissioned; externally peer reviewed.
