# Supplementary material for: Use of menopausal hormone therapy and risk of dementia: nested case-control studies using QResearch and CPRD databases
Source: BMJ. 2021 Sep 30;374:n2182. doi: 10.1136/bmj.n2182 (PMC8479814; doi:10.1136/bmj.n2182)
Supplement: Supplementary file 1 — Web appendix: Supplementary materials [file viny066406.ww1.pdf]

Use of menopausal hormone therapy and risk of dementia: two nested case-control studies in primary care

Supplementary tables

Supplementary Table A Characteristics in cases and controls at least 1 years before the index date across the databases by exposure to MHT/HRT 4
Supplementary Table B **All cases and controls:** Duration of use for different types of MHT/HRT: number of cases and controls and **unadjusted odds ratios** by database and combined results. 6
Supplementary Table C **All cases and controls:** Duration of use for different types and hormones of MHT/HRT, by database 8
Supplementary Table D **All cases and controls:** Duration of use for different doses of hormones and types of application by database 11
Supplementary Table E Other medications prescribed for menopausal women by database and combined analysis 14
Supplementary Table F **All cases and controls:** Gap since the last use of different hormones of MHT/HRT by database. 15
Supplementary Table G **All cases and controls:** Duration of use for different types and hormones of MHT/HRT across the databases, by age at the index date 18
Supplementary Table H **Cases with Alzheimer's disease and with Vascular dementia and controls:** Duration of use for different types and hormones of MHT/HRT and gap since the last use, across the databases 21
Supplementary Table I C**ases and controls registered before their 50th birthday:** Duration of use for different types and hormones of MHT/HRT and gap since the last use, by database 25

Supplementary figures

Supplementary Figure A Proportion of women in different age groups with different types of dementia in GP CPRD records by calendar year...................... 2
Supplementary Figure B Flow-chat for included cases and controls by database 3
Supplementary Figure C Adjusted odds ratios for cases and controls with initiated MHT/HRT at different ages, main analysis and analysis restricted to cases with Alzheimer's disease.. 17
Supplementary Figure D Adjusted odds ratios for linear model of MHT/HRT and tibolone exposures in cases with Alzheimer's disease and their controls ... 24

Supplementary Figure A Proportion of women in different age groups with different types of dementia in GP CPRD records by calendar year

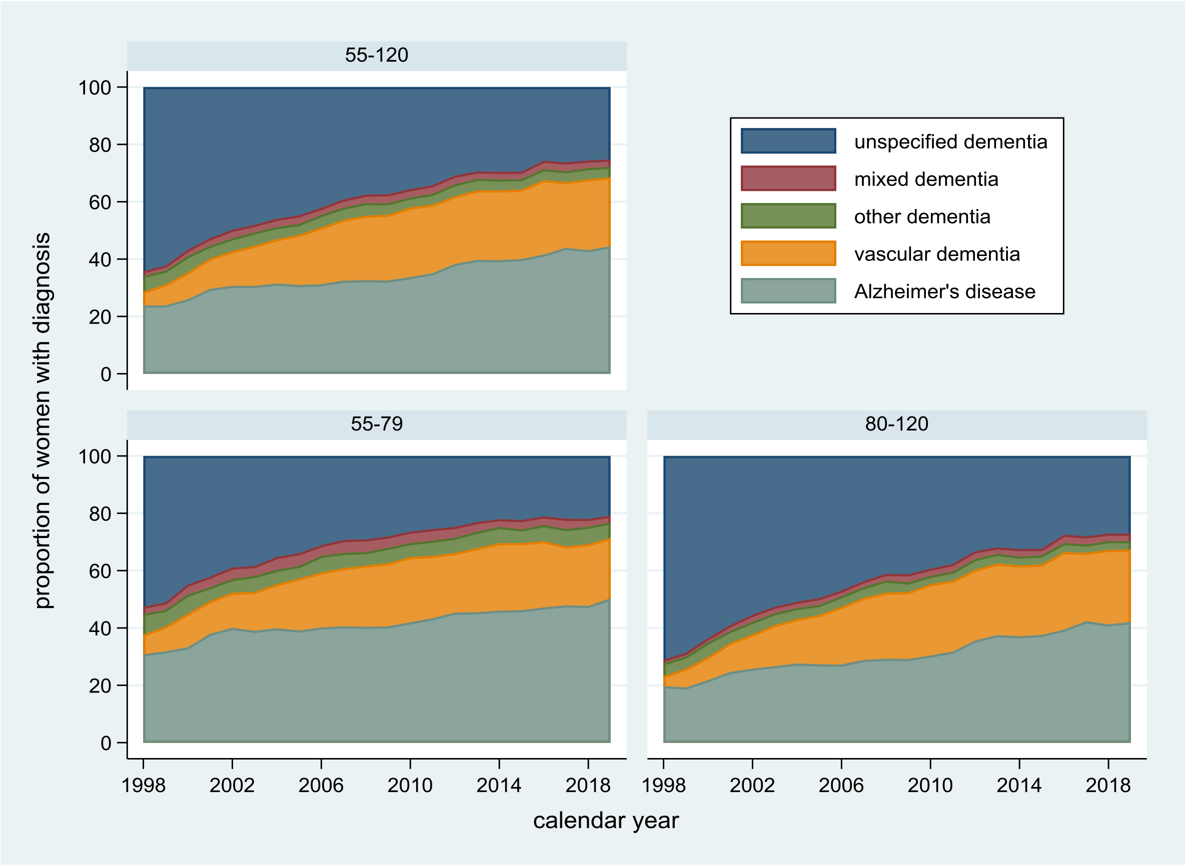

Supplementary Figure B Flow-chat for included cases and controls by database

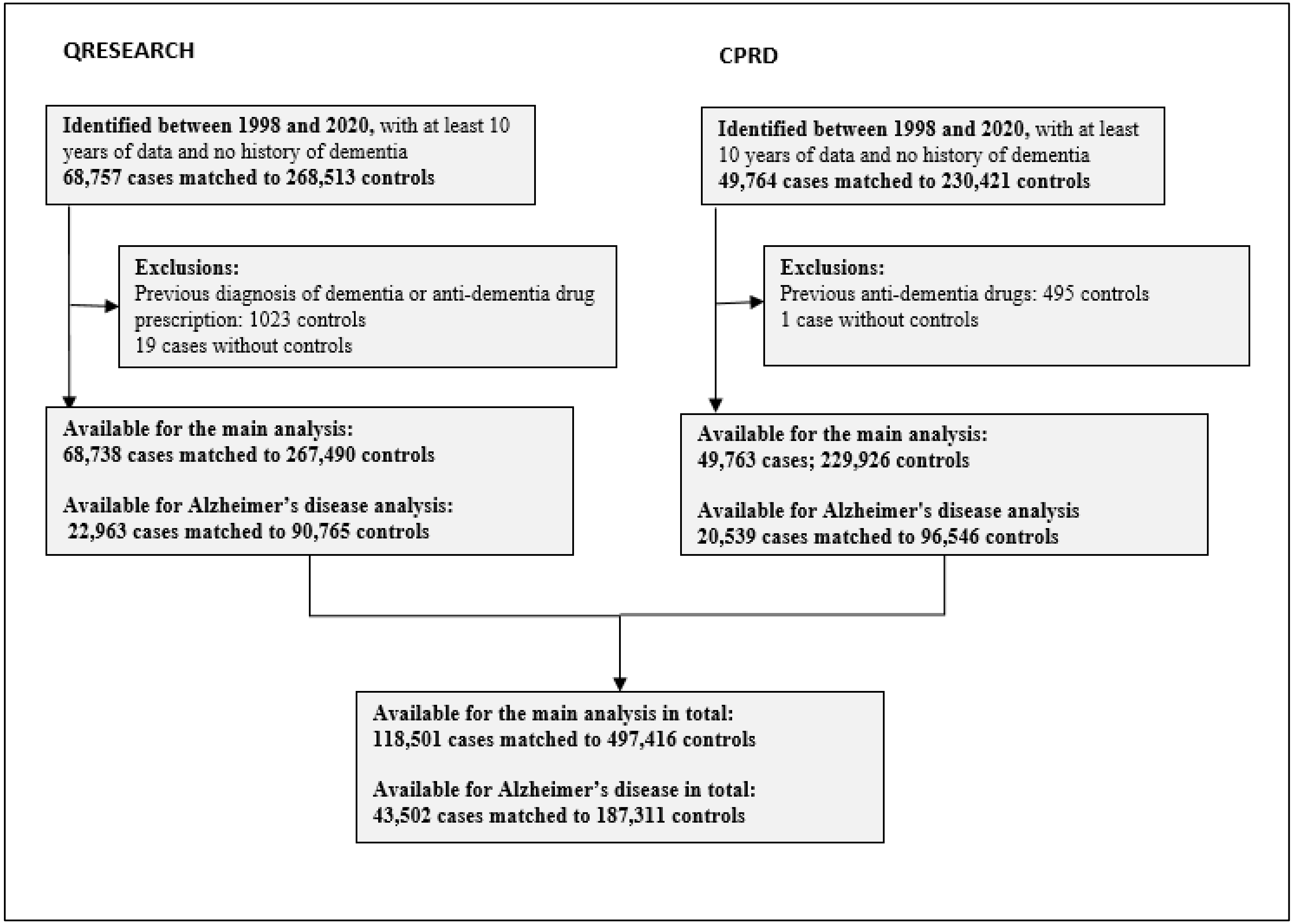

Supplementary Table A Characteristics in cases and controls at least 1 years before the index date across the databases by exposure to MHT/HRT

	**No exposure**		**Oestrogen only**		**Oestrogen-progestogen**	
	Cases; % (N)	Controls; % (N)	Cases; % (N)	Controls; % (N)	Cases; % (N)	Controls; % (N)
Total	102210	428690	6189	25552	10102	43174
**Age in years**						
age (mean, SD)	84.4 (6.6)	83.9 (6.4)	79.1 (6.7)	79.0 (6.7)	77.2 (6.6)	77.2 (6.5)
**Townsend quintile***	*) based on linked cases and controls					
Most affluent	25.9 (21349)	27.3 (91689)	29.1 (1410)	32.4 (6372)	31.3 (2412)	34.2 (10945)
2	24.7 (20362)	25.2 (84681)	26.1 (1266)	26.1 (5132)	25.9 (1995)	26.4 (8443)
3	21.8 (18000)	21.4 (71884)	20.2 (979)	19.6 (3864)	19.8 (1525)	19.0 (6074)
4	17.1 (14120)	16.4 (55214)	15.9 (772)	13.9 (2743)	14.8 (1139)	12.7 (4067)
Most deprived	10.6 (8734)	9.7 (32631)	8.8 (426)	8.0 (1566)	8.3 (636)	7.8 (2500)
**Body mass index** mean (SD)	26.9 (4.9)	27.1 (4.8)	27.1 (4.9)	27.2 (4.7)	26.5 (4.8)	26.6 (4.7)
**Chronic conditions**						
Anxiety	9.2 (9391)	8.2 (35052)	15.3 (947)	13.4 (3420)	16.8 (1693)	14.3 (6156)
Cancer	6.1 (6243)	6.2 (26489)	6.0 (372)	5.7 (1468)	5.7 (575)	5.6 (2411)
Coronary heart disease	11.9 (12171)	10.4 (44494)	13.2 (820)	11.3 (2879)	9.1 (923)	7.2 (3129)
Depression	17.4 (17835)	14.7 (62825)	32.0 (1981)	26.9 (6863)	32.0 (3228)	26.4 (11382)
Diabetes	8.5 (8714)	6.4 (27398)	9.5 (591)	6.3 (1606)	7.1 (717)	4.9 (2127)
Hearing loss	7.3 (7445)	6.7 (28555)	7.6 (472)	7.0 (1795)	7.1 (714)	6.7 (2911)
Hypertension	41.6 (42558)	40.6 (173877)	44.1 (2728)	41.4 (10571)	36.2 (3652)	34.6 (14953)
Parkinson's disease	0.5 (542)	0.2 (985)	0.6 (40)	0.2 (53)	0.8 (82)	0.2 (93)
Stroke	5.4 (5546)	4.2 (17865)	5.5 (339)	4.1 (1054)	4.5 (451)	2.9 (1255)
**Other characteristics**						
early menopause	6.7 (6856)	6.4 (27468)	40.9 (2531)	40.1 (10258)	6.6 (666)	5.7 (2449)
hysterectomy/oophorectomy	17.9 (18252)	17.5 (75175)	88.2 (5461)	89.4 (22854)	13.6 (1378)	12.7 (5496)
menopausal symptoms	9.5 (9668)	9.6 (40981)	36.0 (2225)	37.7 (9621)	48.5 (4902)	48.4 (20904)

**Any use of other medications before the index date**						
anticholinergics	44.2 (45216)	40.5 (173719)	67.4 (4169)	61.8 (15797)	63.3 (6396)	57.8 (24940)
anti-hypertensive drugs	55.8 (57066)	53.4 (228759)	65.1 (4026)	60.4 (15434)	56.2 (5675)	52.8 (22808)
benzodiazepines	12.4 (12691)	11.0 (47230)	23.3 (1444)	20.9 (5343)	22.5 (2275)	19.8 (8536)
statins	21.4 (21885)	18.7 (80235)	31.4 (1946)	25.9 (6628)	25.0 (2522)	21.0 (9077)

Supplementary Table B ***All cases and controls:*** *Duration of use for different types of MHT/HRT: number of cases and controls and* ***unadjusted odds ratios*** *by database and combined results.*

	QResearch		CPRD		Combined	
	N of cases; controls	Unadjusted odds ratio[#] (95% confidence interval)	N of cases; controls	Unadjusted odds ratio[#] (95% confidence interval)	N of cases; controls	unadjusted odds ratio[#] (95% confidence interval)
**OVERALL USE**						
MHT/HRT	9296; 35843	1.04 (1.01 to 1.07)[α]	6995; 32883	1.04 (1.01 to 1.07)	16291; 68726	1.04 (1.02 to 1.06)[β]
Oestrogen only	3569; 13650	1.05 (1.01 to 1.09)	2620; 11902	1.06 (1.01 to 1.11)	6189; 25552	1.05 (1.02 to 1.08)[β]
Combined therapy	5727; 22193	1.04 (1.00 to 1.08)	4375; 20981	1.01 (0.97 to 1.05)	10102; 43174	1.03 (1.00 to 1.05)
**DURATION OF EXPOSURE**						
MHT/HRT						
<1 year	2250; 8296	1.08 (1.03 to 1.14)[α]	2090; 9553	1.05 (1.00 to 1.11)	4340; 17849	1.07 (1.03 to 1.11)[β]
1 to <3 years	1624; 6402	1.02 (0.96 to 1.08)	1471; 6804	1.05 (0.98 to 1.11)	3095; 13206	1.03 (0.99 to 1.07)
3 to <5 years	1286; 5177	0.99 (0.93 to 1.05)	1001; 4910	0.99 (0.92 to 1.06)	2287; 10087	0.99 (0.94 to 1.04)
5 to <10 years	2754; 10725	1.04 (0.99 to 1.08)	1691; 7927	1.04 (0.98 to 1.10)	4445; 18652	1.04 (1.00 to 1.07)
10+ years	1382; 5243	1.07 (1.00 to 1.14)	742; 3689	0.99 (0.91 to 1.07)	2124; 8932	1.04 (0.99 to 1.09)
OESTROGEN ONLY						
<1 year	846; 2948	1.13 (1.05 to 1.23)[α]	762; 3307	1.10 (1.01 to 1.19)	1608; 6255	1.12 (1.06 to 1.18)[β]
1 to <3 years	578; 2247	1.03 (0.94 to 1.13)	518; 2268	1.10 (1.00 to 1.21)	1096; 4515	1.06 (0.99 to 1.14)
3 to <5 years	430; 1833	0.93 (0.83 to 1.03)	348; 1587	1.06 (0.94 to 1.19)	778; 3420	0.99 (0.91 to 1.07)
5 to <10 years	1110; 4222	1.06 (0.99 to 1.13)	663; 3060	1.05 (0.96 to 1.14)	1773; 7282	1.06 (1.00 to 1.11)
10+ years	605; 2400	1.02 (0.93 to 1.11)	329; 1680	0.96 (0.85 to 1.08)	934; 4080	0.99 (0.92 to 1.07)
***OESTROGEN COMBINED with* any progestogen**						
<1 year	1604; 6154	1.04 (0.98 to 1.10)	1514; 7086	1.03 (0.97 to 1.10)	3118; 13240	1.04 (0.99 to 1.08)
1 to <3 years	1125; 4408	1.02 (0.95 to 1.09)	976; 4783	0.99 (0.92 to 1.06)	2101; 9191	1.00 (0.95 to 1.06)
3 to <5 years	879; 3387	1.04 (0.96 to 1.12)	634; 3254	0.94 (0.86 to 1.03)	1513; 6641	1.00 (0.94 to 1.06)
5 to <10 years	1510; 6014	1.02 (0.96 to 1.08)	935; 4390	1.04 (0.96 to 1.12)	2445; 10404	1.02 (0.98 to 1.07)
10+ years	609; 2230	1.11 (1.01 to 1.22)	316; 1468	1.06 (0.93 to 1.20)	925; 3698	1.09 (1.01 to 1.18)
**GAP AFTER THE LAST EXPOSURE**						
OESTROGEN ONLY						

3 to <5 years	523; 2112	0.97 (0.88 to 1.07)	350; 1693	0.98 (0.87 to 1.10)	873; 3805	0.97 (0.90 to 1.05)
5 to <10 years	884; 3133	1.13 (1.05 to 1.22)[α]	616; 2624	1.12 (1.02 to 1.22)	1500; 5757	1.13 (1.06 to 1.19)[β]
10 years or more	2162; 8405	1.03 (0.98 to 1.09)	1654; 7585	1.06 (1.00 to 1.12)	3816; 15990	1.04 (1.01 to 1.08)
***OESTROGEN COMBINED with* any progestogen**						
3 to <5 years	498; 1950	1.02 (0.92 to 1.13)	405; 1609	1.17 (1.04 to 1.31)[α]	903; 3559	1.09 (1.01 to 1.17)
5 to <10 years	1080; 3993	1.10 (1.02 to 1.18)	825; 3489	1.12 (1.04 to 1.22)[α]	1905; 7482	1.11 (1.05 to 1.17)[β]
10 years or more	4149; 16250	1.02 (0.98 to 1.07)	3145; 15883	0.97 (0.92 to 1.01)	7294; 32133	1.00 (0.97 to 1.03)

[#]Odds ratios are based on cases and controls matched by age and practice.

[α] P-value<0.01; [β] P-value<0.001

Supplementary Table C ***All cases and controls:*** *Duration of use for different types and hormones of MHT/HRT, by database*

	QResearch		CPRD		Combined analysis	
	N of cases; controls	Adjusted odds ratio# (95% confidence interval)	N of cases; controls	Adjusted odds ratio# (95% confidence interval)	Combined odds ratio (95% confidence interval)	P-value
**OVERALL USE**						
HRT	9296; 35843	1.00 (0.97 to 1.03)	6995; 32883	0.99 (0.96 to 1.02)	0.99 (0.97 to 1.01)	0.5
Oestrogen only	3569; 13650	0.99 (0.95 to 1.04)	2620; 11902	0.99 (0.94 to 1.04)	0.99 (0.96 to 1.02)	0.5
Combined therapy	5727; 22193	1.00 (0.96 to 1.04)	4375; 20981	1.00 (0.96 to 1.04)	1.00 (0.97 to 1.03)	1
**DURATION OF EXPOSURE**						
**MHT/HRT**						
<1 year	2250; 8296	1.04 (0.99 to 1.09)	2090; 9553	1.02 (0.97 to 1.07)	1.03 (0.99 to 1.07)	0.1
1 to <3 years	1624; 6402	0.98 (0.92 to 1.04)	1471; 6804	1.00 (0.94 to 1.07)	0.99 (0.95 to 1.03)	0.6
3 to <5 years	1286; 5177	0.95 (0.89 to 1.01)	1001; 4910	0.95 (0.88 to 1.02)	0.95 (0.90 to 0.99)	0.03
5 to <10 years	2754; 10725	0.98 (0.94 to 1.03)	1691; 7927	1.00 (0.94 to 1.06)	0.99 (0.95 to 1.03)	0.6
10+ years	1382; 5243	1.00 (0.94 to 1.07)	742; 3689	0.95 (0.87 to 1.03)	0.98 (0.93 to 1.03)	0.4
OESTROGEN ONLY						
<1 year	846; 2948	1.08 (1.00 to 1.17)	762; 3307	1.02 (0.94 to 1.11)	1.05 (0.99 to 1.12)	0.08
1 to <3 years	578; 2247	0.99 (0.90 to 1.09)	518; 2268	1.01 (0.91 to 1.11)	1.00 (0.93 to 1.07)	1
3 to <5 years	430; 1833	0.87 (0.78 to 0.97)	348; 1587	0.98 (0.87 to 1.11)	0.92 (0.85 to 1.00)	0.04
5 to <10 years	1110; 4222	1.00 (0.93 to 1.07)	663; 3060	0.99 (0.90 to 1.08)	0.99 (0.94 to 1.05)	0.8
10+ years	605; 2400	0.94 (0.86 to 1.04)	329; 1680	0.90 (0.80 to 1.02)	0.93 (0.86 to 1.00)	0.05
**Conjugated equine oestrogen**						
<1 year	566; 2080	1.03 (0.94 to 1.14)	456; 1982	1.02 (0.92 to 1.14)	1.03 (0.96 to 1.11)	0.5
1 to <3 years	342; 1375	0.96 (0.85 to 1.09)	284; 1154	1.09 (0.95 to 1.24)	1.02 (0.93 to 1.11)	0.7
3 to <5 years	233; 950	0.92 (0.79 to 1.06)	188; 793	1.07 (0.90 to 1.26)	0.98 (0.88 to 1.09)	0.7
5 to <10 years	557; 2124	0.99 (0.90 to 1.09)	314; 1540	0.94 (0.83 to 1.06)	0.97 (0.90 to 1.05)	0.5
10+ years	291; 1094	0.99 (0.87 to 1.14)	158; 771	0.93 (0.78 to 1.11)	0.97 (0.87 to 1.08)	0.6

**Estradiol**						
<1 year	610; 2224	1.03 (0.93 to 1.13)	622; 2733	1.01 (0.92 to 1.11)	1.02 (0.95 to 1.09)	0.6
1 to <3 years	375; 1447	0.99 (0.88 to 1.12)	342; 1642	0.92 (0.82 to 1.04)	0.96 (0.88 to 1.04)	0.3
3 to <5 years	264; 1106	0.90 (0.78 to 1.03)	192; 997	0.85 (0.73 to 1.00)	0.88 (0.79 to 0.98)	0.02
5 to <10 years	568; 2151	1.00 (0.91 to 1.11)	352; 1566	1.02 (0.90 to 1.15)	1.01 (0.93 to 1.09)	0.8
10+ years	273; 1150	0.89 (0.78 to 1.02)	144; 723	0.93 (0.77 to 1.12)	0.91 (0.81 to 1.01)	0.08
OESTROGEN COMBINED with						
**any progestogen**						
<1 year	1604; 6154	1.00 (0.94 to 1.07)	1514; 7086	1.02 (0.96 to 1.09)	1.01 (0.97 to 1.06)	0.6
1 to <3 years	1125; 4408	0.99 (0.92 to 1.06)	976; 4783	0.98 (0.90 to 1.05)	0.98 (0.93 to 1.03)	0.5
3 to <5 years	879; 3387	1.01 (0.93 to 1.09)	634; 3254	0.92 (0.84 to 1.01)	0.97 (0.92 to 1.03)	0.4
5 to <10 years	1510; 6014	0.98 (0.92 to 1.04)	935; 4390	1.03 (0.95 to 1.11)	1.00 (0.95 to 1.05)	0.9
10+ years	609; 2230	1.05 (0.96 to 1.16)	316; 1468	1.04 (0.91 to 1.18)	1.05 (0.97 to 1.13)	0.2
**Medroxyprogesterone**						
<1 year	740; 2779	1.02 (0.93 to 1.11)	514; 2562	0.94 (0.85 to 1.05)	0.98 (0.92 to 1.05)	0.6
1 to <3 years	424; 1609	1.03 (0.92 to 1.15)	311; 1522	0.96 (0.85 to 1.09)	1.00 (0.92 to 1.09)	1
3 to <5 years	327; 1180	1.07 (0.94 to 1.22)	192; 973	0.95 (0.81 to 1.11)	1.02 (0.92 to 1.13)	0.7
5 years or more	341; 1333	0.98 (0.87 to 1.12)	197; 890	1.05 (0.90 to 1.23)	1.01 (0.92 to 1.11)	0.8
**Levonorgestrel**						
<1 year	1052; 4038	1.01 (0.93 to 1.08)	962; 4429	1.07 (0.99 to 1.15)	1.03 (0.98 to 1.09)	0.2
1 to <3 years	733; 2974	0.96 (0.88 to 1.05)	531; 2608	1.01 (0.91 to 1.12)	0.98 (0.92 to 1.05)	0.6
3 to <5 years	513; 1754	1.15 (1.04 to 1.28)[a]	300; 1515	0.99 (0.87 to 1.13)	1.08 (1.00 to 1.18)	0.05
5 to <10 years	604; 2275	1.03 (0.93 to 1.13)	397; 1855	1.06 (0.94 to 1.19)	1.04 (0.97 to 1.12)	0.3
10+ years	158; 605	1.01 (0.85 to 1.21)	91; 424	1.06 (0.84 to 1.34)	1.03 (0.90 to 1.19)	0.7
**Norethisterone**						
<1 year	1261; 4755	1.00 (0.94 to 1.07)	1163; 5650	0.97 (0.90 to 1.04)	0.99 (0.94 to 1.03)	0.6
1 to <3 years	736; 2838	1.00 (0.92 to 1.09)	551; 2739	0.97 (0.88 to 1.07)	0.99 (0.92 to 1.05)	0.7
3 to <5 years	451; 1827	0.96 (0.86 to 1.07)	333; 1580	0.97 (0.86 to 1.10)	0.97 (0.89 to 1.05)	0.4
5 to <10 years	623; 2405	1.01 (0.92 to 1.11)	354; 1620	1.05 (0.93 to 1.19)	1.03 (0.95 to 1.10)	0.5

10+ years	142; 600	0.89 (0.73 to 1.07)	60; 281	0.98 (0.74 to 1.31)	0.91 (0.78 to 1.07)	0.3
**Dydrogesterone**						
<1 year	176; 716	0.91 (0.77 to 1.09)	145; 674	1.05 (0.87 to 1.27)	0.97 (0.86 to 1.11)	0.7
1 to <3 years	81; 345	0.91 (0.71 to 1.17)	54; 296	0.86 (0.64 to 1.17)	0.89 (0.73 to 1.08)	0.2
3 years or more	50; 194	0.92 (0.67 to 1.26)	27; 172	0.77 (0.50 to 1.17)	0.86 (0.67 to 1.11)	0.2
TIBOLONE						
<1 year	845; 3007	1.06 (0.98 to 1.15)	746; 3602	0.98 (0.90 to 1.06)	1.02 (0.96 to 1.08)	0.5
1 to <3 years	340; 1330	0.99 (0.87 to 1.12)	296; 1396	1.01 (0.89 to 1.15)	1.00 (0.91 to 1.09)	1
3 to <5 years	208; 808	1.00 (0.86 to 1.17)	168; 772	1.03 (0.86 to 1.22)	1.01 (0.90 to 1.14)	0.8
5 to <10 years	298; 1247	0.94 (0.82 to 1.07)	216; 998	1.05 (0.90 to 1.22)	0.98 (0.89 to 1.09)	0.7
10+ years	84; 354	0.91 (0.71 to 1.16)	78; 319	1.18 (0.91 to 1.52)	1.03 (0.86 to 1.22)	0.8

[#]Odds ratios are based on cases and controls matched by age and practice and adjusted for smoking status, body mass index, family history of dementia, medical conditions and events, other medications and contraceptive drugs; [α] P-value<0.01; [β] P-value<0.001

*Supplementary Table D **All cases and controls:** Duration of use for different doses of hormones and types of application by database*

	QResearch		CPRD		Combined analysis	
	N of cases; controls	Adjusted odds ratio# (95% confidence interval)	N of cases; controls	Adjusted odds ratio# (95% confidence interval)	Combined odds ratio (95% confidence interval)	P-value
OESTROGEN ONLY						
**Conjugated equine oestrogen**						
≤0.625mg						
<1 year	482; 1771	1.03 (0.93 to 1.15)	351; 1498	1.05 (0.93 to 1.19)	1.04 (0.96 to 1.13)	0.3
1 to <3 years	273; 1127	0.94 (0.82 to 1.08)	210; 890	1.04 (0.89 to 1.21)	0.98 (0.89 to 1.09)	0.8
3 to <5 years	194; 781	0.93 (0.79 to 1.09)	137; 608	1.01 (0.83 to 1.22)	0.96 (0.85 to 1.09)	0.5
5 to <10 years	431; 1726	0.94 (0.84 to 1.05)	230; 1135	0.94 (0.81 to 1.08)	0.94 (0.86 to 1.03)	0.2
10+ years	211; 817	0.96 (0.82 to 1.13)	112; 550	0.96 (0.78 to 1.19)	0.96 (0.85 to 1.09)	0.6
>0.625mg						
<1 year	84; 309	1.02 (0.80 to 1.31)	105; 484	0.92 (0.74 to 1.14)	0.96 (0.82 to 1.13)	0.6
1 to <3 years	69; 248	1.04 (0.79 to 1.37)	74; 264	1.24 (0.95 to 1.62)	1.14 (0.94 to 1.38)	0.2
3 to <5 years	39; 169	0.84 (0.59 to 1.20)	51; 185	1.24 (0.90 to 1.70)	1.04 (0.82 to 1.32)	0.7
5 to <10 years	126; 398	1.20 (0.98 to 1.48)	84; 405	0.94 (0.74 to 1.19)	1.08 (0.93 to 1.27)	0.3
10+ years	80; 277	1.07 (0.83 to 1.39)	46; 221	0.86 (0.62 to 1.19)	0.99 (0.81 to 1.21)	0.9
**Estradiol**						
≤1mg						
<1 year	570; 2057	1.03 (0.94 to 1.14)	555; 2467	1.00 (0.91 to 1.11)	1.02 (0.95 to 1.09)	0.6
1 to <3 years	337; 1280	1.01 (0.89 to 1.15)	288; 1419	0.90 (0.79 to 1.03)	0.96 (0.88 to 1.05)	0.4
3 to <5 years	215; 982	0.83 (0.71 to 0.96)	154; 814	0.84 (0.71 to 1.01)	0.84 (0.74 to 0.94)	0.002
5 to <10 years	461; 1799	0.97 (0.88 to 1.08)	271; 1245	0.98 (0.86 to 1.13)	0.98 (0.90 to 1.06)	0.6
10+ years	229; 975	0.89 (0.77 to 1.03)	112; 565	0.94 (0.76 to 1.16)	0.91 (0.80 to 1.02)	0.1

>1mg	40; 167	0.92 (0.65 to 1.30)	67; 266	1.09 (0.82 to 1.43)	1.02 (0.82 to 1.26)	0.9
<1 year	38; 167	0.81 (0.57 to 1.17)	54; 223	1.06 (0.78 to 1.44)	0.95 (0.75 to 1.20)	0.7
1 to <3 years	49; 124	1.45 (1.03 to 2.03)	38; 183	0.87 (0.61 to 1.25)	1.14 (0.89 to 1.46)	0.3
3 to <5 years	107; 352	1.13 (0.91 to 1.41)	81; 321	1.13 (0.88 to 1.46)	1.13 (0.96 to 1.34)	0.1
5 to <10 years	44; 175	0.90 (0.64 to 1.27)	32; 158	0.89 (0.60 to 1.31)	0.90 (0.70 to 1.16)	0.4
10+ years						
oral	304; 1135	1.01 (0.89 to 1.16)	273; 1178	1.03 (0.90 to 1.19)	1.02 (0.93 to 1.12)	0.7
<1 year	134; 609	0.83 (0.68 to 1.00)	121; 505	1.08 (0.88 to 1.33)	0.94 (0.81 to 1.08)	0.4
1 to <3 years	117; 424	1.02 (0.83 to 1.26)	61; 328	0.84 (0.63 to 1.11)	0.95 (0.80 to 1.13)	0.6
3 to <5 years	196; 728	1.00 (0.85 to 1.18)	127; 501	1.15 (0.94 to 1.41)	1.06 (0.93 to 1.20)	0.4
5 to <10 years	69; 328	0.77 (0.59 to 1.00)	45; 194	1.10 (0.79 to 1.54)	0.89 (0.72 to 1.09)	0.3
10+ years						
transdermal	455; 1631	1.04 (0.93 to 1.16)	520; 2257	1.03 (0.93 to 1.14)	1.03 (0.96 to 1.11)	0.4
<1 year	282; 1037	1.05 (0.91 to 1.20)	247; 1281	0.85 (0.74 to 0.98)	0.95 (0.86 to 1.05)	0.3
1 to <3 years	164; 699	0.90 (0.75 to 1.07)	145; 705	0.90 (0.75 to 1.08)	0.90 (0.79 to 1.02)	0.1
3 to <5 years	370; 1411	1.01 (0.90 to 1.14)	222; 1072	0.94 (0.81 to 1.09)	0.98 (0.90 to 1.08)	0.7
5 to <10 years	193; 794	0.93 (0.79 to 1.09)	89; 481	0.86 (0.68 to 1.08)	0.91 (0.79 to 1.03)	0.1
10+ years						
OESTROGEN COMBINED with						
**Norethisterone**						
oral	1182; 4501	1.00 (0.93 to 1.07)	1033; 5121	0.96 (0.89 to 1.03)	0.98 (0.93 to 1.03)	0.4
<1 year	595; 2234	1.03 (0.93 to 1.13)	485; 2257	1.04 (0.94 to 1.16)	1.04 (0.96 to 1.11)	0.3
1 to <3 years	344; 1424	0.94 (0.83 to 1.06)	277; 1357	0.95 (0.83 to 1.09)	0.94 (0.86 to 1.03)	0.2
3 to <5 years	503; 1892	1.03 (0.93 to 1.14)	299; 1398	1.04 (0.91 to 1.18)	1.03 (0.95 to 1.12)	0.5
5 to <10 years	94; 390	0.90 (0.71 to 1.13)	53; 229	1.07 (0.79 to 1.45)	0.96 (0.79 to 1.15)	0.6
10+ years						
transdermal	415; 1454	1.07 (0.95 to 1.20)	334; 1622	0.99 (0.88 to 1.13)	1.03 (0.95 to 1.12)	0.5
<1 year	218; 902	0.94 (0.80 to 1.09)	111; 667	0.79 (0.64 to 0.97)	0.88 (0.78 to 1.00)	0.04
1 to <3 years	108; 469	0.90 (0.72 to 1.11)	58; 213	1.24 (0.92 to 1.68)	1.00 (0.84 to 1.19)	1

3 to <5 years	155; 603	1.03 (0.86 to 1.24)	48; 211	1.07 (0.78 to 1.48)	1.04 (0.89 to 1.22)	0.6
5 years or more	482; 1771	1.03 (0.93 to 1.15)	351; 1498	1.05 (0.93 to 1.19)	1.04 (0.96 to 1.13)	0.3

[#]Odds ratios are based on cases and controls matched by age and practice and adjusted for smoking status, body mass index, family history of dementia, medical conditions and events, other medications and contraceptive drugs.

[α] P-value<0.01; [β] P-value<0.001

Supplementary Table E Other medications prescribed for menopausal women by database and combined analysis

	QResearch		CPRD		Combined analysis	
	N of cases; controls	Adjusted odds ratio# (95% confidence interval)	N of cases; controls	Adjusted odds ratio# (95% confidence interval)	Combined odds ratio (95% confidence interval)	P-value
**Oestrogen cream**						
<1 year	7843; 29494	1.01 (0.98 to 1.03)	3650; 15860	1.03 (0.99 to 1.07)	1.01 (0.99 to 1.04)	0.3
1 to <3 years	1549; 5605	1.03 (0.97 to 1.10)	499; 2476	0.89 (0.80 to 0.98)	0.99 (0.94 to 1.05)	0.8
3+ years	777; 3080	0.96 (0.89 to 1.05)	258; 1118	1.02 (0.88 to 1.17)	0.98 (0.91 to 1.05)	0.5
**Vaginal preparations**						
<1 year	3286; 12507	0.98 (0.94 to 1.02)	2780; 12754	0.97 (0.93 to 1.02)	0.98 (0.95 to 1.01)	0.1
1 to <3 years	588; 2353	0.93 (0.85 to 1.02)	470; 2022	1.03 (0.92 to 1.14)	0.97 (0.91 to 1.04)	0.4
3+ years	358; 1420	0.93 (0.82 to 1.04)	233; 1084	0.93 (0.81 to 1.08)	0.93 (0.85 to 1.02)	0.1

#Odds ratios are based on cases and controls matched by age and practice and adjusted for smoking status, body mass index, family history of dementia, medical conditions and events, other medications and contraceptive drugs.

$^{\alpha}$ P-value<0.01; $^{\beta}$ P-value<0.001

*Supplementary Table F **All cases and controls:** Gap since the last use of different hormones of MHT/HRT by database.*

	QResearch		CPRD		Combined analysis	
	N of cases; controls	Adjusted odds ratio# (95% confidence interval)	N of cases; controls	Adjusted odds ratio# (95% confidence interval)	Combined odds ratio (95% confidence interval)	P-value
**MHT/HRT**						
3 to <5 years	1119; 4412	0.96 (0.89 to 1.03)	826; 3662	0.97 (0.89 to 1.05)	0.96 (0.91 to 1.02)	0.2
5 to <10 years	2042; 7414	1.06 (1.01 to 1.12)	1513; 6419	1.06 (1.00 to 1.13)	1.06 (1.02 to 1.11)	0.004
10 years or more	6135; 24017	0.98 (0.95 to 1.02)	4656; 22802	0.97 (0.93 to 1.01)	0.98 (0.95 to 1.00)	0.08
OESTROGEN ONLY						
3 to <5 years	523; 2112	0.92 (0.83 to 1.01)	350; 1693	0.88 (0.78 to 1.00)	0.90 (0.84 to 0.98)	0.01
5 to <10 years	884; 3133	1.08 (0.99 to 1.16)	616; 2624	1.03 (0.94 to 1.13)	1.06 (0.99 to 1.12)	0.07
10 years or more	2162; 8405	0.98 (0.93 to 1.03)	1654; 7585	0.99 (0.94 to 1.06)	0.98 (0.95 to 1.02)	0.5
**Conjugated equine oestrogen**						
3 to <5 years	216; 916	0.87 (0.74 to 1.01)	143; 657	0.93 (0.77 to 1.12)	0.89 (0.79 to 1.00)	0.06
5 to <10 years	425; 1500	1.07 (0.96 to 1.20)	291; 1182	1.06 (0.93 to 1.21)	1.07 (0.98 to 1.16)	0.1
10 years or more	1348; 5207	0.99 (0.93 to 1.06)	966; 4401	1.00 (0.92 to 1.08)	1.00 (0.95 to 1.05)	0.9
**Estradiol**						
3 to <5 years	315; 1248	0.94 (0.83 to 1.07)	212; 1075	0.84 (0.72 to 0.98)	0.90 (0.82 to 0.99)	0.04
5 to <10 years	532; 1885	1.08 (0.98 to 1.19)	389; 1671	1.03 (0.92 to 1.16)	1.06 (0.98 to 1.14)	0.1
10 years or more	1243; 4945	0.95 (0.89 to 1.02)	1051; 4915	0.97 (0.90 to 1.05)	0.96 (0.91 to 1.01)	0.1
OESTROGEN COMBINED with						
**any progestogen**						
3 to <5 years	498; 1950	0.98 (0.89 to 1.09)	405; 1609	1.09 (0.97 to 1.23)	1.03 (0.95 to 1.11)	0.5
5 to <10 years	1080; 3993	1.05 (0.98 to 1.13)	825; 3489	1.09 (1.00 to 1.18)	1.07 (1.01 to 1.13)	0.02
10 years or more	4149; 16250	0.99 (0.95 to 1.03)	3145; 15883	0.97 (0.92 to 1.01)	0.98 (0.95 to 1.01)	0.2
**Medroxyprogesterone**						
3 to <5 years	138; 609	0.87 (0.72 to 1.05)	117; 481	1.04 (0.84 to 1.29)	0.94 (0.82 to 1.09)	0.4
5 to <10 years	396; 1425	1.09 (0.97 to 1.23)	280; 1184	1.05 (0.92 to 1.21)	1.08 (0.98 to 1.18)	0.1

10 years or more	1298; 4867	1.03 (0.96 to 1.11)	817; 4282	0.93 (0.85 to 1.00)	0.99 (0.94 to 1.04)	0.6
Levonorgestrel						
3 to <5 years	100; 427	0.91 (0.73 to 1.14)	77; 294	1.08 (0.83 to 1.41)	0.98 (0.82 to 1.16)	0.8
5 to <10 years	332; 1157	1.13 (1.00 to 1.29)	213; 907	1.05 (0.89 to 1.23)	1.10 (0.99 to 1.21)	0.07
10 years or more	2628; 10062	1.02 (0.96 to 1.07)	1991; 9630	1.03 (0.98 to 1.10)	1.02 (0.99 to 1.06)	0.2
**Norethisterone**						
3 to <5 years	279; 1003	1.07 (0.93 to 1.22)	227; 887	1.07 (0.91 to 1.25)	1.07 (0.96 to 1.18)	0.2
5 to <10 years	579; 2250	1.00 (0.90 to 1.10)	491; 2033	1.08 (0.97 to 1.20)	1.03 (0.96 to 1.11)	0.4
10 years or more	2355; 9172	0.98 (0.93 to 1.04)	1743; 8950	0.94 (0.89 to 1.00)	0.97 (0.93 to 1.00)	0.08
**Dydrogesterone**						
3 to <5 years	60; 283	0.73 (0.55 to 0.97)	72; 308	1.07 (0.81 to 1.40)	0.89 (0.73 to 1.09)	0.3
5 to <10 years	247; 972	0.97 (0.84 to 1.13)	154; 834	0.90 (0.75 to 1.08)	0.94 (0.84 to 1.06)	0.3
10 years or more						
TIBOLONE						
3 to <5 years	159; 652	0.94 (0.79 to 1.13)	136; 580	1.03 (0.85 to 1.25)	0.98 (0.86 to 1.12)	0.8
5 to <10 years	336; 1328	0.98 (0.87 to 1.11)	300; 1163	1.19 (1.04 to 1.35)	1.07 (0.98 to 1.17)	0.1
10 years or more	1280; 4766	1.03 (0.96 to 1.10)	1068; 5344	0.97 (0.90 to 1.04)	1.00 (0.95 to 1.05)	0.9

[#]Odds ratios are based on cases and controls matched by age and practice and adjusted for smoking status, body mass index, family history of dementia, medical conditions and events, other medications and contraceptive drugs.

Supplementary Figure C Adjusted odds ratios for cases and controls with initiated MHT/HRT at different ages, main analysis and analysis restricted to cases with Alzheimer's disease

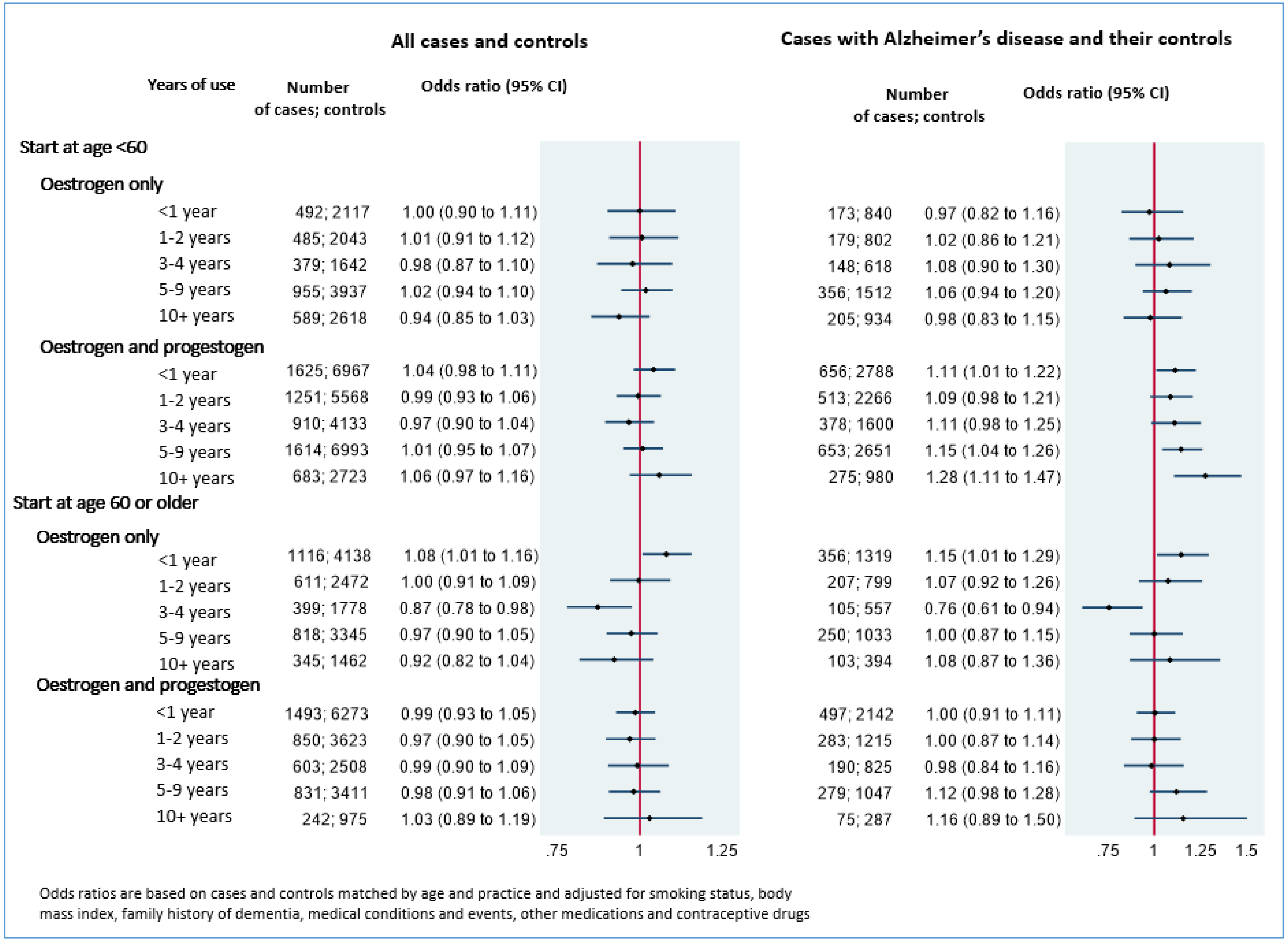

*Supplementary Table G **All cases and controls:** Duration of use for different types and hormones of MHT/HRT across the databases, by age at the index date*

	Age between 55 and 79		Age 80 and older	
	N of cases; controls	Combined adjusted odds ratio[#] (95% confidence interval)	N of cases; controls	Combined adjusted odds ratio[#] (95% confidence interval)
Total number of women	32010; 139845		86491; 357571	
**OVERALL USE**				
MHT/HRT	9587; 40604	0.99 (0.96 to 1.02)	6704; 28122	0.98 (0.95 to 1.01)
Oestrogen only	3203; 13269	0.95 (0.91 to 1.00)	2986; 12283	0.99 (0.95 to 1.04)
Combined therapy	6384; 27335	1.02 (0.98 to 1.06)	3718; 15839	0.97 (0.93 to 1.02)
**DURATION OF EXPOSURE**				
**MHT/HRT**				
<1 year	2258; 9475	1.02 (0.97 to 1.08)	2082; 8374	1.03 (0.98 to 1.09)
1 to <3 years	1932; 8107	1.01 (0.96 to 1.07)	1163; 5099	0.94 (0.88 to 1.00)
3 to <5 years	1448; 6345	0.95 (0.89 to 1.01)	839; 3742	0.92 (0.85 to 1.00)
5 to <10 years	2751; 11530	1.00 (0.95 to 1.05)	1694; 7122	0.97 (0.91 to 1.02)
10+ years	1198; 5147	0.94 (0.87 to 1.01)	926; 3785	1.01 (0.93 to 1.09)
OESTROGEN ONLY				
<1 year	689; 2714	1.01 (0.93 to 1.11)	919; 3541	1.06 (0.99 to 1.15)
1 to <3 years	593; 2479	0.96 (0.87 to 1.06)	503; 2036	1.02 (0.92 to 1.12)
3 to <5 years	444; 1900	0.91 (0.81 to 1.02)	334; 1520	0.89 (0.79 to 1.00)
5 to <10 years	1000; 4009	0.99 (0.91 to 1.07)	773; 3273	0.96 (0.89 to 1.04)
10+ years	477; 2167	0.85 (0.76 to 0.94)[α]	457; 1913	0.98 (0.88 to 1.09)
per year of use		0.989 (0.982 to 0.996)[β]		
**Conjugated equine oestrogen**				
<1 year	494; 1963	1.00 (0.90 to 1.12)	528; 2099	1.03 (0.93 to 1.13)
1 to <3 years	363; 1389	1.07 (0.94 to 1.21)	263; 1140	0.94 (0.82 to 1.08)
3 to <5 years	236; 956	0.99 (0.85 to 1.15)	185; 787	0.94 (0.80 to 1.11)
5 to <10 years	463; 1940	0.95 (0.85 to 1.06)	408; 1724	0.96 (0.86 to 1.08)
10+ years	221; 945	0.89 (0.76 to 1.04)	228; 920	1.01 (0.87 to 1.17)

**Estradiol**				
<1 year	579; 2411	0.97 (0.88 to 1.07)	653; 2546	1.04 (0.95 to 1.14)
1 to <3 years	388; 1786	0.86 (0.76 to 0.96)	329; 1303	1.07 (0.94 to 1.21)
3 to <5 years	266; 1218	0.85 (0.74 to 0.98)	190; 885	0.87 (0.74 to 1.02)
5 to <10 years	546; 2121	1.01 (0.91 to 1.12)	374; 1596	0.96 (0.85 to 1.07)
10+ years	214; 1010	0.83 (0.71 to 0.97)	203; 863	0.97 (0.83 to 1.14)
OESTROGEN COMBINED with				
**any progestogen**				
<1 year	1802; 7778	1.02 (0.96 to 1.08)	1316; 5462	1.00 (0.94 to 1.07)
1 to <3 years	1415; 5967	1.04 (0.97 to 1.11)	686; 3224	0.88 (0.81 to 0.96)[a]
3 to <5 years	1006; 4422	0.99 (0.91 to 1.06)	507; 2219	0.95 (0.86 to 1.05)
5 to <10 years	1596; 6870	1.01 (0.95 to 1.08)	849; 3534	0.98 (0.91 to 1.06)
10+ years	565; 2298	1.03 (0.94 to 1.14)	360; 1400	1.07 (0.95 to 1.21)
**Medroxyprogesterone**				
<1 year	767; 3335	0.98 (0.90 to 1.07)	487; 2006	1.00 (0.90 to 1.12)
1 to <3 years	512; 2139	1.05 (0.94 to 1.16)	223; 992	0.93 (0.80 to 1.09)
3 to <5 years	342; 1420	1.04 (0.91 to 1.17)	177; 733	1.00 (0.85 to 1.19)
5 years or more	319; 1344	1.00 (0.88 to 1.14)	219; 879	1.02 (0.88 to 1.19)
**Levonorgestrel**				
<1 year	1335; 5560	1.04 (0.97 to 1.12)	679; 2907	1.00 (0.91 to 1.09)
1 to <3 years	828; 3625	0.98 (0.90 to 1.07)	436; 1957	0.96 (0.86 to 1.08)
3 to <5 years	537; 2173	1.08 (0.97 to 1.19)	276; 1096	1.10 (0.96 to 1.27)
5 to <10 years	618; 2633	1.00 (0.91 to 1.10)	383; 1497	1.10 (0.98 to 1.24)
10+ years	143; 601	1.04 (0.86 to 1.26)	106; 428	1.03 (0.83 to 1.28)
**Norethisterone**				
<1 year	1496; 6616	0.97 (0.91 to 1.03)	928; 3789	1.01 (0.93 to 1.09)
1 to <3 years	898; 3731	1.05 (0.97 to 1.13)	389; 1846	0.87 (0.77 to 0.97)
3 to <5 years	536; 2354	0.97 (0.88 to 1.07)	248; 1053	0.97 (0.84 to 1.12)
5 to <10 years	642; 2690	1.05 (0.95 to 1.15)	335; 1335	1.01 (0.89 to 1.14)

10+ years	138; 526	1.03 (0.85 to 1.25)	64; 355	0.73 (0.56 to 0.96)
**Dydrogesterone**				
<1 year	234; 1017	0.97 (0.83 to 1.13)	87; 373	0.97 (0.76 to 1.23)
1 to <3 years	93; 485	0.83 (0.66 to 1.04)	42; 156	1.09 (0.76 to 1.55)
3 years or more	57; 278	0.89 (0.66 to 1.20)	20; 88	0.80 (0.48 to 1.33)
TIBOLONE				
<1 year	845; 3545	1.02 (0.94 to 1.10)	746; 3064	1.03 (0.95 to 1.13)
1 to <3 years	347; 1521	0.97 (0.86 to 1.10)	289; 1205	1.04 (0.91 to 1.18)
3 to <5 years	214; 883	1.04 (0.89 to 1.21)	162; 697	0.98 (0.82 to 1.17)
5 to <10 years	258; 1175	0.93 (0.81 to 1.07)	256; 1070	1.04 (0.90 to 1.19)
10+ years	76; 292	1.15 (0.89 to 1.50)	86; 381	0.95 (0.75 to 1.20)

[#]Odds ratios are based on cases and controls matched by age and practice and adjusted for smoking status, body mass index, family history of dementia, medical conditions and events, other medications and contraceptive drugs; $^{\alpha}$ P-value<0.01; $^{\beta}$ P-value<0.001

Supplementary Table H ***Cases with Alzheimer's disease and with Vascular dementia and controls:*** *Duration of use for different types and hormones of MHT/HRT and gap since the last use, across the databases*

	Alzheimer's disease			Vascular dementia	
	N of cases; controls	Combined adjusted odds ratio# (95% confidence interval)	P-value	N of cases; controls	Combined adjusted odds ratio# (95% confidence interval)
Total number of women	43502; 187311			28493; 121571	
**OVERALL USE**					
MHT/HRT	7332; 30129	1.05 (1.01 to 1.09)	0.005	3773; 16248	0.95 (0.91 to 0.99)
Oestrogen only	2612; 10861	1.01 (0.96 to 1.07)	0.7	1525; 6086	0.96 (0.90 to 1.03)
Combined therapy	4720; 19268	1.08 (1.03 to 1.12)	<0.001	2248; 10162	0.95 (0.89 to 1.00)
**DURATION OF EXPOSURE**					
**MHT/HRT**					
<1 year	1901; 7661	1.09 (1.03 to 1.15)	0.003	1010; 4329	0.96 (0.89 to 1.03)
1 to <3 years	1379; 5914	1.01 (0.95 to 1.08)	0.7	719; 3099	0.94 (0.86 to 1.03)
3 to <5 years	1028; 4424	0.99 (0.92 to 1.07)	0.8	536; 2364	0.92 (0.83 to 1.02)
5 to <10 years	2047; 8264	1.06 (1.00 to 1.12)	0.04	1027; 4384	0.96 (0.89 to 1.04)
10+ years	977; 3866	1.08 (1.00 to 1.17)	0.05	481; 2072	0.94 (0.85 to 1.05)
OESTROGEN ONLY					
<1 year	665; 2558	1.11 (1.02 to 1.22)	0.02	417; 1536	1.03 (0.92 to 1.16)
1 to <3 years	463; 1962	0.99 (0.89 to 1.10)	0.9	272; 1072	0.98 (0.85 to 1.13)
3 to <5 years	318; 1472	0.89 (0.79 to 1.01)	0.08	203; 808	0.96 (0.82 to 1.13)
5 to <10 years	772; 3184	1.01 (0.93 to 1.11)	0.7	412; 1708	0.93 (0.82 to 1.04)
10+ years	394; 1685	0.98 (0.87 to 1.10)	0.8	221; 962	0.88 (0.76 to 1.03)
**Conjugated equine oestrogen**					
<1 year	407; 1672	1.03 (0.92 to 1.15)	0.6	268; 986	1.04 (0.90 to 1.20)
1 to <3 years	264; 1055	1.05 (0.91 to 1.21)	0.5	161; 601	1.03 (0.86 to 1.24)
3 to <5 years	165; 727	0.94 (0.79 to 1.11)	0.5	115; 404	1.09 (0.87 to 1.35)
5 to <10 years	371; 1588	1.00 (0.89 to 1.12)	1.0	217; 870	0.97 (0.83 to 1.14)
10+ years	179; 751	1.01 (0.85 to 1.20)	0.9	100; 428	0.89 (0.71 to 1.12)

**Estradiol**					
<1 year	527; 2086	1.08 (0.98 to 1.19)	0.1	323; 1178	1.06 (0.93 to 1.21)
1 to <3 years	304; 1434	0.89 (0.78 to 1.01)	0.08	155; 692	0.86 (0.72 to 1.04)
3 to <5 years	188; 923	0.85 (0.72 to 1.00)	0.05	119; 492	0.95 (0.77 to 1.18)
5 to <10 years	409; 1637	1.03 (0.91 to 1.15)	0.7	204; 865	0.90 (0.77 to 1.06)
10+ years	189; 777	0.99 (0.84 to 1.17)	0.9	102; 457	0.86 (0.69 to 1.08)
OESTROGEN COMBINED with					
**any progestogen**					
<1 year	1412; 5836	1.07 (1.00 to 1.14)	0.05	682; 3188	0.90 (0.82 to 0.99)
1 to <3 years	972; 4186	1.03 (0.96 to 1.12)	0.4	462; 2133	0.91 (0.81 to 1.02)
3 to <5 years	708; 2982	1.04 (0.96 to 1.14)	0.3	342; 1529	0.96 (0.84 to 1.09)
5 to <10 years	1179; 4621	1.11 (1.04 to 1.20)	0.003	553; 2470	0.96 (0.87 to 1.07)
10+ years	449; 1643	1.19 (1.06 to 1.33)	0.002	209; 842	1.09 (0.93 to 1.28)
**Medroxyprogesterone**					
<1 year	603; 2328	1.08 (0.98 to 1.18)	0.1	284; 1244	1.00 (0.87 to 1.15)
1 to <3 years	368; 1458	1.06 (0.94 to 1.19)	0.4	146; 729	0.90 (0.75 to 1.09)
3 to <5 years	245; 984	1.05 (0.91 to 1.22)	0.5	107; 471	1.01 (0.81 to 1.26)
5 years or more	278; 1025	1.11 (0.96 to 1.27)	0.1	102; 499	0.90 (0.72 to 1.13)
**Levonorgestrel**					
<1 year	926; 3827	1.04 (0.96 to 1.13)	0.3	443; 2002	0.99 (0.89 to 1.11)
1 to <3 years	583; 2515	1.00 (0.91 to 1.10)	1.0	278; 1282	0.97 (0.84 to 1.12)
3 to <5 years	406; 1470	1.18 (1.05 to 1.32)	0.006	179; 791	1.03 (0.87 to 1.22)
5 to <10 years	450; 1826	1.05 (0.94 to 1.17)	0.4	238; 985	1.10 (0.94 to 1.28)
10+ years	125; 433	1.25 (1.02 to 1.54)	0.03	55; 244	1.03 (0.76 to 1.39)
**Norethisterone**					
<1 year	1146; 4688	1.02 (0.95 to 1.10)	0.6	516; 2413	0.91 (0.82 to 1.01)
1 to <3 years	601; 2543	1.01 (0.92 to 1.11)	0.9	278; 1295	0.93 (0.81 to 1.07)
3 to <5 years	360; 1524	1.00 (0.88 to 1.12)	0.9	185; 800	0.99 (0.83 to 1.17)

5 to <10 years	470; 1757	1.14 (1.03 to 1.27)	0.02	233; 963	1.06 (0.91 to 1.23)
10+ years	94; 392	0.98 (0.78 to 1.23)	0.9	48; 189	1.11 (0.80 to 1.54)
**Dydrogesterone**					
<1 year	170; 634	1.09 (0.91 to 1.30)	0.4	60; 315	0.81 (0.61 to 1.09)
1 to <3 years	62; 289	0.90 (0.68 to 1.19)	0.5	32; 127	1.17 (0.78 to 1.77)
3 years or more	40; 162	0.98 (0.68 to 1.40)	0.9	18; 86	0.84 (0.49 to 1.42)
TIBOLONE					
<1 year	726; 2881	1.07 (0.98 to 1.17)	0.1	381; 1531	1.06 (0.94 to 1.20)
1 to <3 years	285; 1215	1.00 (0.87 to 1.14)	1.0	145; 652	1.02 (0.84 to 1.23)
3 to <5 years	170; 695	1.04 (0.87 to 1.23)	0.7	74; 379	0.89 (0.69 to 1.15)
5 to <10 years	242; 936	1.12 (0.97 to 1.30)	0.1	118; 552	0.96 (0.78 to 1.18)
10+ years	82; 286	1.24 (0.96 to 1.59)	0.10	45; 158	1.23 (0.87 to 1.74)
**GAP AFTER THE LAST EXPOSURE**					
OESTROGEN ONLY					
3 to <5 years	370; 1573	0.94 (0.84 to 1.06)	0.3	197; 809	0.95 (0.80 to 1.12)
5 to <10 years	617; 2449	1.04 (0.94 to 1.14)	0.5	396; 1423	1.08 (0.96 to 1.22)
10 years or more	1625; 6839	1.02 (0.96 to 1.08)	0.6	932; 3854	0.92 (0.85 to 1.00)
***OESTROGEN COMBINED with* any progestogen**					
3 to <5 years	442; 1543	1.19 (1.06 to 1.33)	0.002	183; 813	0.92 (0.78 to 1.10)
5 to <10 years	934; 3275	1.23 (1.14 to 1.34)	<0.001	418; 1784	1.01 (0.90 to 1.13)
10 years or more	3344; 14450	1.03 (0.98 to 1.08)	0.3	1647; 7565	0.93 (0.87 to 1.00)

[#]Odds ratios are based on cases and controls matched by age and practice and adjusted for smoking status, body mass index, family history of dementia, medical conditions and events, other medications and contraceptive drugs. No statistically significant associations were found for Vascular dementia.

Supplementary Figure D Adjusted odds ratios for linear model of MHT/HRT and tibolone exposures in cases with Alzheimer's disease and their controls

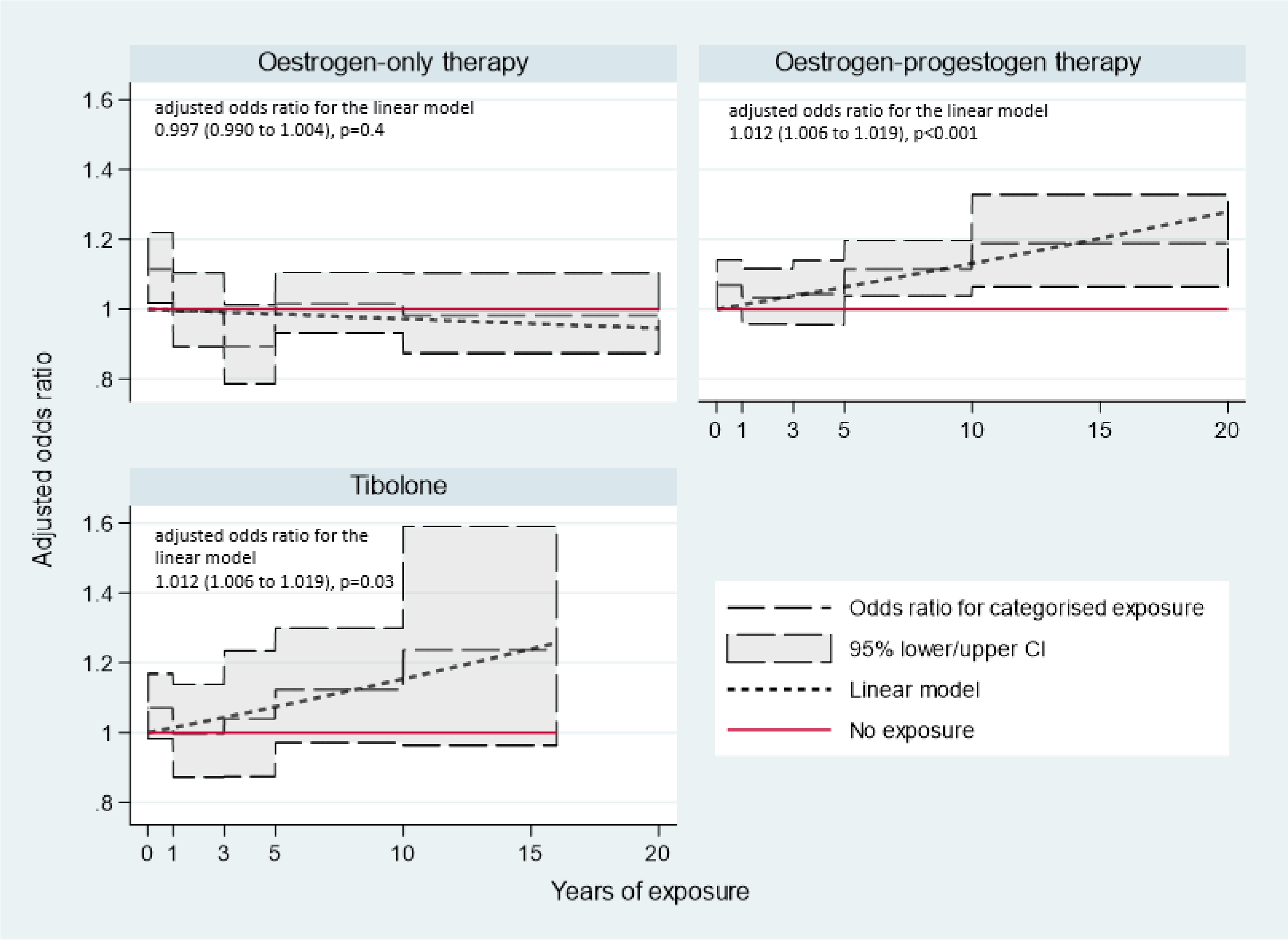

Odds ratios are based on cases and controls matched by age and practice and adjusted for smoking status, body mass index, family history of dementia, medical conditions and events, other medications and contraceptive drugs

*Supplementary Table I C**ases and controls registered before their 50th birthday:** Duration of use for different types and hormones of MHT/HRT and gap since the last use, by database*

	**QResearch**		**CPRD**		**Combined analysis**	
	N of cases; controls	Adjusted odds ratio[#] (95% confidence interval)	N of cases; controls	Adjusted odds ratio[#] (95% confidence interval)	Combined odds ratio (95% confidence interval)	P-value
Total number of women	942; 3071		1689; 8329			
**OVERALL USE**						
HRT	499; 1636	0.98 (0.82 to 1.16)	836; 3814	1.31 (1.16 to 1.48)[β]	1.18 (1.07 to 1.31)	0.001
Oestrogen only	141; 426	1.07 (0.79 to 1.45)	212; 1035	1.18 (0.91 to 1.53)	1.13 (0.93 to 1.38)	0.2
Combined therapy	358; 1210	0.96 (0.80 to 1.17)	624; 2779	1.40 (1.22 to 1.61)[β]	1.23 (1.10 to 1.37)	<0.001
**DURATION OF EXPOSURE**						
**HRT**						
<1 year	91; 286	0.90 (0.68 to 1.20)	200; 920	1.28 (1.07 to 1.54)[α]	1.16 (0.99 to 1.35)	0.06
1 to <3 years	84; 251	1.07 (0.79 to 1.45)	193; 847	1.43 (1.18 to 1.73)[β]	1.32 (1.12 to 1.55)	<0.001
3 to <5 years	69; 247	0.85 (0.63 to 1.17)	137; 599	1.38 (1.11 to 1.72)[α]	1.18 (0.99 to 1.41)	0.07
5 to <10 years	166; 565	0.99 (0.78 to 1.25)	216; 1025	1.36 (1.13 to 1.64)[α]	1.20 (1.04 to 1.39)	0.01
10+ years	89; 287	1.02 (0.76 to 1.36)	90; 423	1.37 (1.06 to 1.79)	1.20 (0.99 to 1.46)	0.07
OESTROGEN ONLY						
<1 year	21; 51	1.22 (0.67 to 2.21)	37; 212	0.93 (0.62 to 1.41)	1.02 (0.73 to 1.43)	0.9
1 to <3 years	21; 52	1.18 (0.66 to 2.12)	44; 201	1.33 (0.89 to 1.97)	1.28 (0.92 to 1.78)	0.1
3 to <5 years	15; 49	0.99 (0.51 to 1.93)	33; 147	1.30 (0.83 to 2.04)	1.20 (0.83 to 1.74)	0.3
5 to <10 years	50; 171	0.94 (0.63 to 1.42)	67; 307	1.33 (0.93 to 1.89)	1.15 (0.88 to 1.50)	0.3
10+ years	34; 103	1.14 (0.71 to 1.82)	31; 168	1.16 (0.73 to 1.82)	1.15 (0.83 to 1.59)	0.4
***OESTROGEN COMBINED with* any progestogen**						
<1 year	82; 262	0.89 (0.66 to 1.21)	195; 824	1.42 (1.17 to 1.72)[β]	1.24 (1.05 to 1.46)	0.01
1 to <3 years	73; 229	1.06 (0.76 to 1.48)	155; 679	1.44 (1.17 to 1.77)[β]	1.32 (1.10 to 1.58)	0.002
3 to <5 years	55; 204	0.84 (0.59 to 1.19)	101; 459	1.37 (1.07 to 1.75)	1.16 (0.94 to 1.42)	0.2
5 to <10 years	104; 362	1.02 (0.78 to 1.34)	129; 633	1.32 (1.05 to 1.65)	1.19 (1.00 to 1.41)	0.05
10+ years	44; 153	0.95 (0.65 to 1.39)	44; 184	1.55 (1.08 to 2.21)	1.23 (0.95 to 1.59)	0.1

**GAP AFTER THE LAST EXPOSURE**						
MHT/HRT						
3 to <5 years	65; 198	1.29 (0.91 to 1.83)	147; 740	1.23 (1.00 to 1.52)	1.25 (1.04 to 1.50)	0.02
5 to <10 years	100; 356	0.92 (0.70 to 1.23)	208; 939	1.36 (1.12 to 1.64)$^{\alpha}$	1.21 (1.03 to 1.41)	0.02
10 years or more	334; 1082	0.93 (0.76 to 1.12)	481; 2135	1.41 (1.21 to 1.64)$^{\beta}$	1.20 (1.07 to 1.35)	0.003
OESTROGEN ONLY						
3 to <5 years	19; 63	1.16 (0.63 to 2.12)	38; 225	0.91 (0.60 to 1.39)	0.99 (0.70 to 1.39)	0.9
5 to <10 years	25; 99	0.76 (0.45 to 1.29)	56; 279	1.09 (0.76 to 1.57)	0.97 (0.72 to 1.31)	0.8
10 years or more	97; 264	1.13 (0.81 to 1.59)	118; 531	1.34 (1.00 to 1.80)	1.25 (1.00 to 1.56)	0.05
***OESTROGEN COMBINED with* any progestogen**						
3 to <5 years	41; 117	1.50 (0.99 to 2.28)	93; 448	1.34 (1.03 to 1.72)	1.38 (1.11 to 1.72)	0.004
5 to <10 years	70; 231	1.05 (0.76 to 1.46)	144; 621	1.46 (1.18 to 1.82) $^{\beta}$	1.32 (1.10 to 1.59)	0.003
10 years or more	247; 862	0.87 (0.70 to 1.07)	387; 1710	1.41 (1.19 to 1.66) $^{\beta}$	1.17 (1.03 to 1.33)	0.02

[#] Odds ratios are based on cases and controls matched by age and practice and adjusted for smoking status, body mass index, family history of dementia, medical conditions and events, other medications and contraceptive drugs; $^{\alpha}$ P-value<0.01; $^{\beta}$ P-value<0.001
